# Salt lakes of La Mancha (Central Spain): A hot spot for tiger beetle (Carabidae, Cicindelinae) species diversity

**DOI:** 10.3897/zookeys.561.6042

**Published:** 2016-02-08

**Authors:** Paula C. Rodríguez-Flores, Jorge Gutiérrez-Rodríguez, Ernesto F. Aguirre-Ruiz, Mario García-París

**Affiliations:** 1 Museo Nacional de Ciencias Naturales, MNCN–CSIC. José Gutiérrez Abascal, 2. 28006 Madrid. España; 2 Fundación Global Nature, Calle Real, 48, Local A. Las Rozas. Madrid. 28231

**Keywords:** Coleoptera, Habitat selection, Phenology, Behaviour, Conservation, Salt marshes, Lakes

## Abstract

The tiger beetle assemblage of the wetlands of La Mancha (central Spain) comprises nine species: *Calomera littoralis littoralis*, *Cephalota maura maura*, *Cephalota circumdata imperialis*, *Cephalota dulcinea*, *Cicindela campestris campestris*, *Cicindela maroccana*, *Cylindera paludosa*, *Lophyra flexuosa flexuosa*, and *Myriochila melancholica melancholica*. This assemblage represents the largest concentration of tiger beetles in a single 1º latitude / longitude square in Europe. General patterns of spatial and temporal segregation among species are discussed based on observations of 1462 specimens registered during an observation period of one year, from April to August. The different species of Cicindelini appear to be distributed over space and time, with little overlapping among them. Three sets of species replace each other phenologically as the season goes on. Most of the species occupy drying or dried salt lakes and salt marshes, with sparse vegetation cover. Spatial segregation is marked in terms of substrate and vegetation use. *Calomera littoralis* and *Myriochila melancholica* have been observed mainly on wet soils; *Cephalota circumdata* on dry open saline flats; *Cephalota dulcinea* and *Cylindera paludosa* in granulated substrates with typical halophytic vegetation; *Cephalota maura* is often present in man-modified areas. *Cephalota circumdata* and *Cephalota dulcinea* are included as species of special interest in the list of protected species in Castilla–La Mancha. Conservation problems for the Cicindelini assemblage arise from agricultural activities and inadequate use of sport vehicles. Attempts at restoring the original habitat, supressing old semi-industrial structures, may affect the spatial heterogeneity of the lakes, and have an effect on Cicindelinae diversity.

## Introduction

The geomorphology of the Iberian Peninsula has been modelled through a long and complex paleogeographic history, including the ancient emergence of the western territories, complex Miocene reorganization of the tectonic plates with active orogenic periods and incorporation of a large portion of the Betic–Rifean massif, and the effect of the Pleistocene glacial periods (López Martínez 1989, Barbadillo et al. 1997, Hewitt 2000, Cavazza et al. 2004, Gutiérrez et al. 2008). As a result of this complex history, the diversification of the Iberian biota has attained such a level as to be considered part of one of the 25 world hotspots of biodiversity (Myers et al. 2000). The complexity of the Iberian faunal assemblages is enhanced by the persistence of representatives of ancient lineages (Machordom and Doadrio 2001, Martínez-Solano et al. 2006, Albert et al. 2007, Gonçalves et al. 2007), together with the settlement of Holocene recent immigrants from the African Continent (Carranza et al. 2004, Recuero et al. 2007).

The Central Iberian Plateau, is an almost flat elevated plain which occupies most of the central area of the Peninsula. The Iberian Plateau, is delimited by mountain chains of different height all around its perimeter, and crossed transversally by the Central System Mountain Chain, which divides it into two sub-regions: a northern one, mostly conformed by the Duero River Basin, located in the Spanish Autonomous Region of Castilla – León, and a southern one crossed by the Tagus and Guadiana river systems, in the Spanish Autonomous Region of Castilla – La Mancha (Cirujano 1980, de la Peña and Marfil 1986, Comín and Alonso 1988, Alonso 1998, Gutiérrez et al. 2008, de Luis et al. 2010). In general terms, the biological diversity of the Iberian Plateau is relatively limited with regard to the surrounding mountainous areas. However, the paleogeographic history of the Plateau has many singularities with regard to all other Iberian structural regions. The Iberian Plateau was covered by large endorheic lakes along most of the Tertiary (Macau and Riba 1965, Plans 1969, Ordóñez et al. 1987, 1991, López Martínez 1989). Those lakes were drained during the Pliocene, along with the formation of the current main river systems that crossed the plateau in an east-west direction, leaving thick layers of gypsum soils (Gutiérrez et al. 2008). Because of the relatively high elevation of the Plateau, 650–800 m, the effect of the glaciations was relatively strong, allowing for the development of a typical taiga vegetation, interrupted by large swamps which covered most of the southern Plateau during the coldest periods (Zagwijn 1996, Ravazzi 2002). Some of those swamps and small lakes are still present in the central areas of the southern Plateau, particularly in La Mancha region. Many of the small lakes, endorheic and temporary, are shallow and highly saline, and when dried present a flat surface covered by salt crusts (de la Peña and Marfil 1986, Comín and Alonso 1988, Florín et al. 1993).

Flat, sun exposed areas surrounded by halophytic vegetation, create a typical habitat for tiger beetles (Cicindelinae) (Vives and Vives 1978b, Rueda and Montes 1987, Hoback et al. 2000, Pearson and Vogler 2001, Jaskuła 2007, 2011, 2015). La Mancha lakes, isolated in the centre of the Plateau, are diverse, not only in salt concentration and depth, but also in vegetation cover and land use around them, offering a wide range of microhabitats suitable for tiger beetle activity. Tiger beetles are active predators, spread over most of the world. In temperate regions most species are terrestrial and hunt actively, running after their prey on sun exposed relatively flat surfaces, such as beaches, lake and river shores, dirt roads, forest clearings, and high elevation denuded areas. Larvae are also predators that hide in self-excavated vertical tunnels, in the same habitats where adult activity takes place (Pearson 1988, Cassola and Pearson 2000, Pearson and Vogler 2001).

The tiger beetle fauna of the Iberian Peninsula is considered well-known in relation to the knowledge on other taxonomic groups. Serrano (2013) indicated the presence of 22 species of Cicindelinae in the Iberian Peninsula, some of them represented by more than one subspecies. However, a large portion of the current knowledge has been generated recently, including the confirmation of the specific differentiation of *Cicindela lagunensis* Gautier des Cottes, 1872, *Cicindela iberica* Mandl, 1935, *Cicindela lusitanica* Mandl, 1935, and *Cicindela hybrida* Linnaeus, 1758 (Matalin 1998, Cardoso and Vogler 2005, Cardoso et al. 2009), the long-awaited description of *Cephalota dulcinea* López, Rosa & Baena, 2006, the analyses of the genetic differentiation of some Iberian species of *Cephalota* (López-López and Galián 2010) and their conservation (Diogo et al. 1999), and the first confirmed reports for Spain of *Cephalota luctuosa* (Dejean, 1831) (Werner 1992) and *Calomera lunulata* (Fabricius, 1781) (López-Pérez and García 2007, López-Pérez 2010). All these new discoveries suggest that we are still far from having a complete knowledge of the Iberian Fauna of tiger beetles. Taxonomic characterization of complex species groups, detailed knowledge on geographic distribution at the local level and basic information on reproductive biology and habitat use are still lacking for most of the species of tiger beetles along most of the Iberian geography.

Previous reports on the Cicindelinae of La Mancha lacustrine areas (Vives and Vives 1978a, Rueda and Montes 1987, Ortíz et al. 1988; Serrano et al. 1990, Lencina et al. 1991, 2001, Andújar et al. 2002, 2009) indicate that the region hosts a particularly large number of tiger beetles when compared to other European regions (Jaskuła 2011). The relevance of salt marshes for tiger beetle diversity and their conservation has been reported often, but the description of *Cephalota dulcinea*, an endemic tiger beetle of La Mancha, restricted to a few salt lakes (López et al. 2006), enhanced the interest on the region as a singular spot for Cicindelinae diversity.

In this study, we monitored, from April to August, the presence and activity of tiger beetles in more than 30 small lakes, salt marshes, and gypsum flats in La Mancha region. Most of these locations (27) were monitored as part of a Life Project: *La Mancha Wetlands* (LIFE+10 NAT/ES/000563), aimed at restoring the *espartal* or *albardinal* (*Lygeum spartum* (L.) Kunth) grasslands and salt flats, by reclaiming Mediterranean salt steppes (*Limonietalia*), designated as a priority habitat (1510) by Habitats Directive 92/43/EEC, and other halophytic formations in the La Mancha wetlands SCI and SPA zones. Most of the locations studied are protected under the laws of the Autonomous Region of Castilla – La Mancha (Spain), and included in the Nature 2000 European Union conservation program (http://ec.europa.eu/environment/nature/natura2000/db_gis/).

Our main objective was to determine the relevance of the lacustrine environments of La Mancha region for tiger beetle diversity and conservation. During the study we found that the number of species inhabiting the region had been underestimated. In comparison to previously published European locality records, La Mancha region harbours the largest number of species of tiger beetles present in a single square (one degree latitude x longitude). This finding prompted us to undertake the following tasks: (1) inventory the tiger beetle species inhabiting each lake or marsh, (2) identify the phenology for each species during the period of study, (3) gather information on general landscape use for each species, and (4) evaluate threats and possible conservation needs, based on observed abundances and observations on human activities around the lakes.

## Methods

### The study area

**Figure 1.**
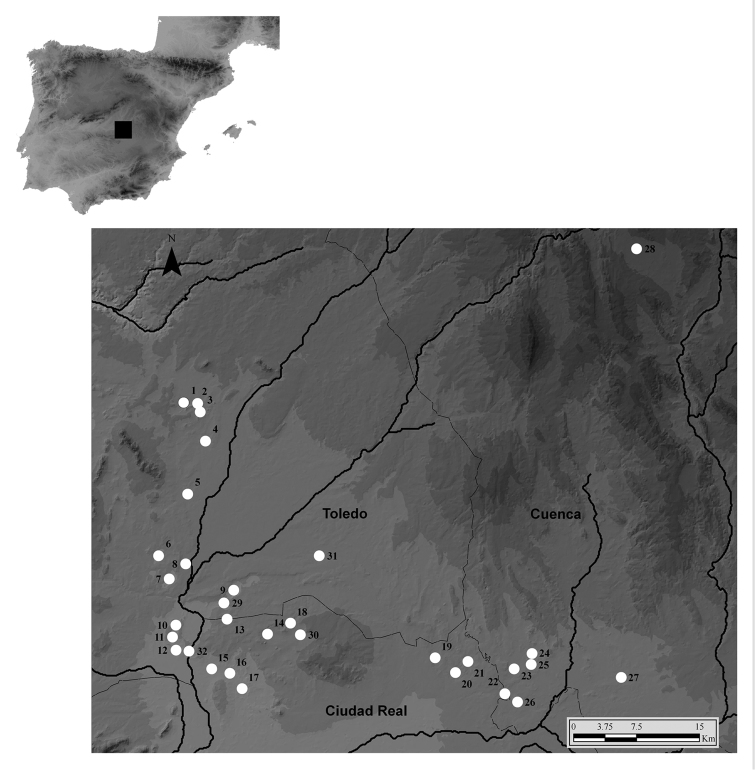
Map of the Iberian Peninsula showing the location of the study area. The inset shows a detail of the 27 marshes and small lakes included in the Life Project “La Mancha Wetlands” (**1–27**), and some other monitored marshes and lakes (**28–32**). Numbers represent the sampled areas: TOLEDO: Lillo: **1** Laguna de El Longar **2** Laguna del Altillo Chica **3** Laguna del Altillo Grande **4** Laguna de la Albardiosa; Miguel Esteban: **31** La Laguna de Miguel Esteban; Villacañas: **5** Laguna Larga **6** Laguna de Tirez **7** Laguna de Peña Hueca; Quero: **8** Laguna del Taray **9** Laguna Grande **29** Cerro de San Cristóbal; Villafranca de Los Caballeros: **10** Laguna Chica **11** Laguna Grande **12** Laguna de la Sal **32** Gigüela marshes. CIUDAD REAL: Alcázar de San Juan: **13** Laguna de los Carros **14** Laguna de Pajares/los Pájaros **15** Laguna de las Yeguas **16** Laguna del Camino de Villafranca **17** Laguna de la Veguilla; Campo de Criptana: **18** Laguna de Salicor **30** Arroyo de San Marcos; Pedro Muñoz: **19** Laguna del Retamar **20** Laguna del Pueblo/de la Vega **21** Laguna de Navalafuente **22** Laguna de Alcahozo. CUENCA: Mota del Cuervo: **23** Laguna de Manjavacas **24** Laguna de Sánchez Gómez **25** Laguna de la Dehesilla; Las Mesas/Las Pedroñeras: **26** Laguna de Alcahozo Chico **27** Laguna del Taray Chico. El Hito: **28** Laguna del Hito.

The study was carried on the 27 marshes and small lakes included in the Life Project *La Mancha Wetlands*, all located in the traditional demarcation of La Mancha, which include portions of Ciudad Real, Cuenca, and Toledo provinces (Fig. 1; Table 1). Some additional salt marshes, ancient channels, and gypsum flats, located in possible corridor areas between lakes were also studied. Amongst those, the gypsum flats and channels of Cerro San Cristóbal in Quero (Toledo), and salt marshes of the Gigüela River in Quero and Villafranca de los Caballeros (Toledo) were also monitored. Some larger lakes not included in the Life Project, as Laguna del Hito (Cuenca), or La Laguna in Miguel Esteban (Toledo), were also included in the study (Fig. 1; Table 1). Despite their current protection, and their healthy appearance, most of the small lakes and swamps have suffered important modifications and historical aggressions of all kinds, from being ploughed in dry years, to being partially filled with urban waste, and flooded with residual waters when located near villages. Some of the lakes most visited by bird-watchers (La Veguilla in Alcázar de San Juan), were artificially created for human uses, less than four decades ago.

**Table 1.** Localities monitored, geographic coordinates and sampling dates for each locality.

Wetland	Town and province	Coordinates	Date
Laguna de El Longar	Lillo (Toledo)	39°42'8.00"N, 3°19'17.86"W	15-VI-2012, 17-VI-2012, 13-IV-2014, 23-V-2014, 13-VI-2014, 11-VII-2014, 2-VIII-2014, 9-VIII-2014, 23-VIII-2014
Laguna del Altillo Chico	Lillo (Toledo)	39°42'4.57"N, 3°18'11.38"W	23-V-2014, 7-VI-2014, 13-VI-2014, 2-VIII-2014
Laguna del Altillo Grande	Lillo (Toledo)	39°41'33.10"N, 3°18'1.03"W	17-V-2014, 23-V-2014, 7-VI-2014, 13-VI-2014
Laguna de la Albardiosa	Lillo (Toledo)	39°39'38.75"N, 3°17'32.35"W	23-V-2014
Laguna Larga	Villacañas (Toledo)	39°36'17.45"N, 3°19'1.58"W	26-IV-2014, 17-V-2014, 23-V-2014, 13-VI-2014, 20-VI-2014, 27-VI-2014, 9-VIII-2014, 23-VIII-2014
Laguna de Tirez	Villacañas (Toledo)	39°32'6.85"N, 3°21'26.73"W	17-VI-2012, 13-IV-2014, 17-V-2014, 20-VI-2014, 27-VI-2014, 2-VIII-2014, 23-VIII-2014, 24-X-2014
Laguna de Peña Hueca	Villacañas (Toledo)	39°30'50.58"N, 3°20'24.98"W	13-IV-2014, 26-IV-2014, 17-V-2014, 23-V-2014, 13-VI-2014, 27-VI-2014, 11-VII-2014, 2-VIII-2014, 9-VIII-2014, 23-VIII-2014, 24-X-2014
Laguna del Taray	Quero (Toledo)	39°31'48.62"N, 3°18'59.79"W	26-IV-2014, 7-VI-2014, 21-VI-2014
Laguna Grande	Quero (Toledo)	39°29'59.25"N, 3°15'12.42"W	26-IV-2014, 24-V-2014, 21-VI-2014, 28-VI-2014, 12-VII-2014
Laguna Chica	Villafranca de los Caballeros (Toledo)	39°27'51.61"N, 3°19'56.61"W	26-IV-2014, 20-VI-2014, 27-VI-2014
Laguna Grande	Villafranca de los Caballeros (Toledo)	39°27'3.42"N, 3°20'14.67"W	26-IV-2014, 20-VI-2014, 27-VI-2014, 11-VII-2014, 2-VIII-2014, 9-VIII-2014
Laguna de la Sal	Villafranca de los Caballeros (Toledo)	39°26'14.71"N, 3°19'54.51"W	26-IV-2014, 24-V-2014, 11-VII-2014
Laguna de los Carros	Alcázar de San Juan / Quero	39°28'12.39"N, 3°15'45.30"W	8-VI-2012, 26-IV-2014, 24-V-2014, 21-VI-2014
Laguna de Pajares	Alcázar de San Juan (Ciudad Real)	39°27'15.47"N, 3°12'20.83"W	25-IV-2014, 17-V-2014, 7-VI-2014
Laguna de las Yeguas	Alcázar de San Juan (Ciudad Real)	39°25'0.58"N, 3°16'58.85"W	26-IV-2014, 17-V-2014, 24-V-2014, 20-VI-2014, 27-VI-2014, 11-VII-2014, 27-VII-2014, 2-VIII-2014, 9-VIII-2014
Laguna del Camino de Villafranca	Alcázar de San Juan (Ciudad Real)	39°24'52.88"N, 3°15'30.63"W	26-IV-2014, 11-VII-2014, 27-VII-2014, 2-VIII-2014, 9-VIII-2014
Laguna de la Veguilla	Alcázar de San Juan (Ciudad Real)	39°23'45.37"N, 3°14'24.14"W	26-IV-2014, 11-VII-2014, 8-VIII-2014, 9-VIII-2014
Laguna de Salicor	Campo de Criptana (Ciudad Real)	39°28'0.24"N, 3°10'24.78"W	8-VI-2012, 13-IV-2014, 17-V-2014, 6-VI-2014, 7-VI-2014 , 28-VI-2014, 8-VIII-2014
Laguna de Retamar	Pedro Muñoz (Ciudad Real)	39°25'42.06"N, 2°58'20.98"W	25-IV-2014, 21-VI-2014
Laguna del Pueblo	Pedro Muñoz (Ciudad Real)	39°24'39.52"N, 2°56'47.20"W	25-IV-2014, 6-VI-2014, 28-VI-2014, 12-VII-2014, 8-VIII-2014
Laguna de Navalafuente	Pedro Muñoz (Ciudad Real)	39°25'31.02"N, 2°55'44.17"W	25-IV-2014
Laguna de Alcahozo	Pedro Muñoz (Ciudad Real)	39°23'27.46"N, 2°52'31.21"W	25-IV-2014, 16-V-2014, 6-VI-2014, 21-VI-2014 , 8-VIII-2014
Laguna de Manjavacas	Mota del Cuervo (Cuenca)	39°25'0.21"N, 2°51'55.06"W	25-IV-2014, 16-V-2014, 21-VI-2014, 28-VI-2014, 12-VII-2014, 8-VIII-2014
Laguna de Sánchez Gómez	Mota del Cuervo (Cuenca)	39°25'55.79"N, 2°50'22.26"W	25-IV-2014, 16-V-2014, 6-VI-2014, 21-VI-2014, 28-VI-2014, 8-VIII-2014,
Laguna de la Dehesilla	Mota del Cuervo (Cuenca)	39°25'20.28"N, 2°50'25.51"W	16-V-2014, 6-VI-2014, 20-VI-2014, 8-VIII-2014
Laguna de Alcahozo chico	Mota del Cuervo (Cuenca)	39°23'26.99"N, 2°52'32.64"W	25-IV-2014, 16-V-2014, 6-VI-2014, 12-VII-2014
Laguna del Taray Chico	Las Mesas / Las Pedroñeras (Cuenca)	39°24'30.19"N, 2°42'55.98"W	25-IV-2014, 16-V-2014,
Laguna de El Hito	Montalbo / El Hito (Cuenca)	39°52'4.22"N, 2°41'34.02"W	15-VI-2014
Cerro San Cristóbal	Quero (Toledo)	39°29'16.10’’N 3°15'58.62’’W	8-VI-2012 , 24-V-2014, 21-VI-2014
Arroyo de San Marcos	Campo de Criptana (Ciudad Real)	39°27'13.66’’N 3°09'37.29’’W	8-VI-2012, 17-V-2014,
La Laguna	Miguel Esteban (Toledo)	39°32'19.15’’N 3°08'03.70’’W	12-VII-2014
Gigüela marshes	Villafranca de los Caballeros (Toledo)	39°26'09.36’’N 3°18'52.00’’W	24-V-2014

The temporary lakes of La Mancha are a group of wetlands spread across the territory of the Upper Guadiana Basin. According to Jerez García (2010), these wetlands are characterised by the presence of sulphates (magnesium sulphate) and, to a lesser extent, calcium carbonates and chloride (sodium chloride). These water bodies are endorheic and, during summer, water can evaporate completely, thus rendering them saline or hyper-saline.

These singularities – fluctuating saline lakes or hyper-saline lakes, with salt concentrations that can exceed those of seawater, and vegetation adapted to fairly restrictive conditions – make these wetlands and their surroundings particularly interesting for biodiversity conservation. These areas harbour a large number of endemic or endangered halophytic plants [i.e. *Limonium carpetanicum* Erben, *Limonium dichotomum* (Cav.) Kuntze Revis., *Limonium squarrosum* Erben, *Gypsophila tomentosa* L., *Lygeum spartum*, *Microcnemum coralloides* (Loscos & J.Pardo) Font Quer] and aquatic plants [*Lamprothamnium papulosum* (K.Wallroth) J.Groves, *Tolypella salina* R. Corillion, *Althenia orientalis* (Tzvelev) P.García-Murillo and Talavera, and *Ruppia drepanensis* Tineo ex Guss.] (Cirujano and Medina 2002, Cirujano et al. 2014).

The lakes of La Mancha and the protective steppes that surround them, provide extensive ecosystem services (Florín and Montes 1999) and function as habitats for many species of endangered invertebrates and vertebrates (de la Cruz 2009). However, the saline wetlands of La Mancha are as important as they are unknown throughout Spain and the rest of Europe. In the last decade, the increase in cultivated land in the area, together with the construction of ditches and canals to prevent waterlogging, has contributed to the destruction of much of the natural vegetation surrounding the cuvettes (Aguirre et al. 2013).

### Sampling and monitoring strategy

Tiger beetle sampling and monitoring was designed to cover the period in which most saline lakes are totally or partially dry, from April to September. Monitoring in 2014 consisted in ½ to 1 hour walks for visual census in favourable habitats within marshes and lakes, including halophytic vegetation, shores of lakes, denuded salt flats, dirt roads with or without saline surfaces, draining channel banks, and margins of cultivated fields. Walks were carried out by one or two people (occasionally more) (MG-P, PCR-F, and collaborators mentioned in the acknowledgments). In particular situations (e.g. behavioural observations) walks took longer than 1 hour. At a first stage, we established transects to evaluate abundance and population density, but populations moved or changed locations to nearby areas along the monitoring season, rendering transects unsuitable. As a consequence, sampling effort and dates changed across patches, so statistical analyses of tiger beetle densities were no longer adequate. This particular situation is quite different from that observed in other studies (Mazzei et al. 2014), in which specimens were located along defined transects during the entire period of survey.

Most species of cicindelids in the region are easy to identify *de visu* without the need of seizing the specimens. Only *Cicindela campestris* and *Cicindela maroccana* are difficult to differentiate at a distance. In those cases, and in any other particularly difficult situation, specimens were captured with an entomological hand-net. Captured specimens were released on site, except for a few per location that were kept as vouchers or reference, or for future confirmation of identification based on molecular analyses (*Cicindela campestris* and *Cicindela maroccana* difficult cases). Specimens captured are preserved dry or in ethanol at the Museo Nacional de Ciencias Naturales (MNCN–CSIC). Walks started no earlier than 11 AM and ended around sunset (from 18:30 to 21:30 PM depending on the season).

Each sampled area was visited between 1 and 11 times during the period of study (Table 1), the number varying in accordance with its potential to harbour undetected species in previous visits, or to follow individual site changes in species phenology. Day samplings were complemented with occasional night observations to detect resting places or possible nocturnal activity. Larval sampling was not undertaken. Additional surveys were performed by car in peripheral areas. These surveys consisted of low speed searches along dirt roads (about 180 km), with the objective of detecting populations inhabiting areas far from the larger marshes and lakes. Additional observations from a 2012 preliminary survey (Villacañas, Lillo, Alcázar de San Juan, and Campo de Criptana) (MG-P), and from local collaborators in Alcázar de San Juan (Pablo Pichaco), were also taken into account.

Sampling was not adequately designed to monitor species with late winter and early spring activity, such as *Cicindela campestris*, *Cicindela maroccana*, and possibly *Lophyra flexuosa* that could be already active around completely water- filled lakes. Therefore, presence and abundances recorded for these three species are not representative of their actual temporal and spatial yearly distribution.

Unpredictable climate changes have a strong influence on the phenology and patterns of activity of tiger beetles. Consequently, in these current years in which rain and temperature patterns are often erratic, the results obtained can only be considered representative of the season studied. Climate changes may have a particularly strong effect on the species that develop most of their activity in dry lake basins, which may suffer strong delays or acceleration during the drying period.

Each specimen was geo-referenced and the general types of soil and vegetation were recorded. Categories used for soil structure were: salt crust (coarse or sheet salt layers on lake basins), salt patches in trails (small salt flats outcropping on patches on trails), granulated salty soils (areas with a thick layer of non-crystalline salty soils, usually gritty or dusty); compact soils in flat trails, compact soils in the banks of channels, saprobe shores (soils formed by decomposed organic matter in lake basins with heavy avian populations), and muds (wet soils with lower salt concentration) (Table 2).

**Table 2.** Number of specimens of tiger beetles found on each of the different substrate and vegetation types considered. See text for details.

Soil type / Species	*Calomera littoralis*	*Cephalota maura*	*Cephalota dulcinea*	*Cephalota circumdata*	*Cicindela campestris*	*Cylindera paludosa*	*Myriochila melancholica*
Salt crust	227	15	5	82		1	
Granulated salty soils	116	12	279	19	45	41	
Salt patches in trails		20	153			24	
Trails		11	87		7		
Banks		27			13	1	
Sapropel shores	52						
Mud	166	11			13		35
**Vegetation type / Species**							
**Denuded areas**	274	63	113	83	12	4	10
**Halophytic prairies**							
open	132	15	289	18	21	28	
dense	2	11			33	8	
**Non halophytic prairies**							
open	47				5		
**Albardinal**							
open			122			26	
**Shore hidrophytic vegetation**							
open	106	1			3	1	25
dense		6			4		

Categories used for vegetation cover were: denuded (areas without vegetation) or vegetated. We split vegetated areas into halophytic and non-halophytic prairies. Within the halophytic category, two main groups were selected: *albardinal*, open or dense, dominated by *Lygeum spartum* (considered apart because of its ecological singularities and because it is the main target of the conservation and restoration programs); and other halophytic prairies, open or dense (which include all types of prairies located on salt soils, from the temporarily flooded edges of the saline basins, to the never flooded margin of the lakes. Their vegetation include vegetal communities dominated mainly by *Salicornia ramossisima* J.Woods, *Limonium* Mill., *Suaeda vera* Forssk. ex J.F.Gmel., *Salsola vermiculata* L., *Microcnemum coralloides* (Loscos & J. Pardo) Font Quer, *Plantago maritima* L., *Suaeda splendens* (Pourr.) Gren. & Godr., *Frankenia pulverulenta* L., *Hordeum marinum* Huds., *Sphenopus divaricatus* (Gouan) Reichenb., *Lolium rigidum* (Gaudin) Weiss ex Nyman, and *Bolboschoenus maritimus* (L.) Palla at the shore (Cirujano 1981) (Table 2).

The non-halophytic category, open or dense, includes vegetation not necessarily associated with salty soils, either on relatively dry soils [dominated in the area by *Scirpus holoschoenus* L., *Cirsium monspessulanus* (L.) Hill., *Plantago major* L., *Trifolium fragiferum* L., *Cynodon dactylon* (L.) Pers.] (Cirujano 1981), or on wet soils [formed by hydrophytic communities dominated by *Scirpus littoralis* Shrad. *Phragmites australis* (Cav.) Trin. ex Steud., *Typha angusifolia* L., etc.] (Cirujano 1980) (Table 2).

## Results

**Figure 2.**
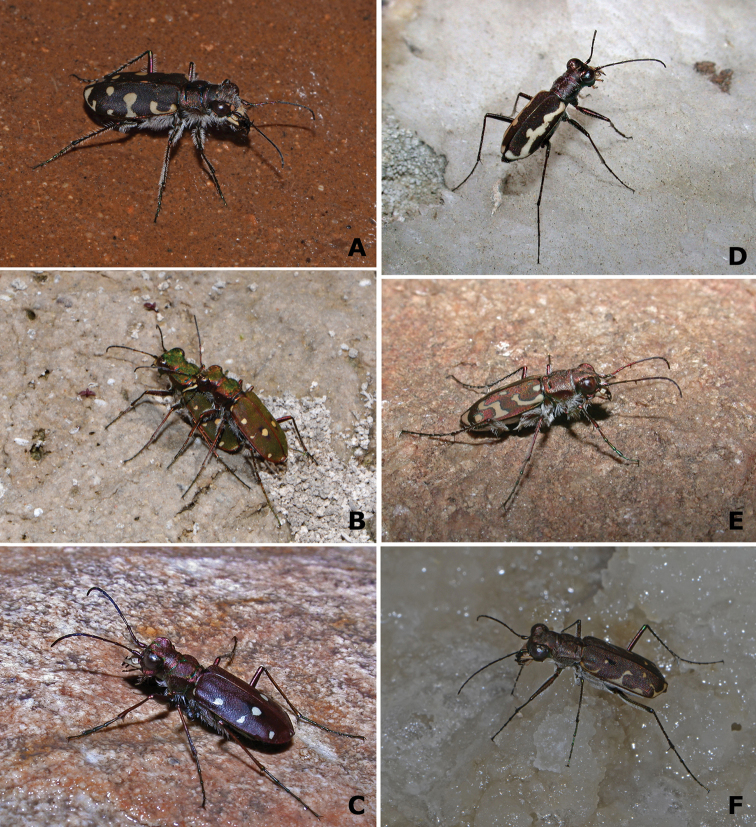
Representative specimens of tiger beetles from La Mancha wetlands **A** *Calomera littoralis littoralis* (La Dehesilla, Cuenca) **B** *Cicindela campestris campestris* (La Dehesilla, Cuenca) **C** *Cicindela maroccana* (La Sal, Toledo) **D** *Cylindera paludosa* (El Longar, Toledo) **E** *Lophyra flexuosa flexuosa* (El Pardo, Madrid) **F** *Myriochila melancholica melancholica* (Tirez, Toledo). Photographs by MG-P.

**Figure 3.**
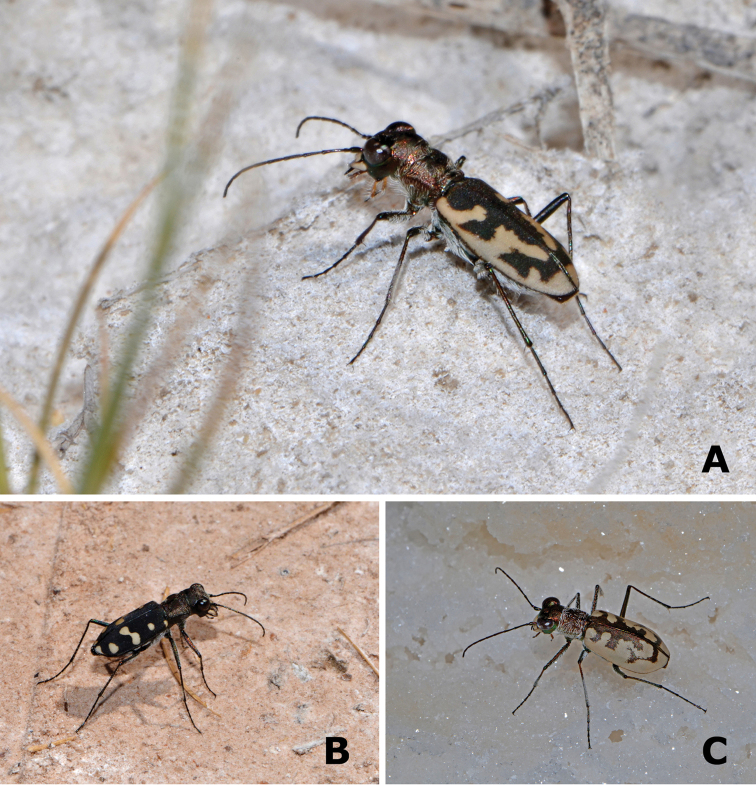
Representative specimens of *Cephalota* from La Mancha wetlands. **A** Cephalota (Taenidia) dulcinea (Sánchez Gómez, Cuenca) **B** Cephalota (Cassolaia) maura (Arroyo San Marcos, Ciudad Real) **C** Cephalota (Taenidia) circumdata imperialis (Tirez, Toledo). Photographs by MG-P.

Presence and number of tiger beetles observed at each locality visited is recorded in Table 3 and Suppl. material 1. We observed a total of 1462 specimens corresponding to 9 species of Cicindelinae (Figs 2–3): *Calomera littoralis littoralis* (Fabricius, 1787), Cephalota (Cassolaia) maura maura (Linnaeus, 1758), Cephalota (Taenidia) circumdata imperialis Klug, 1834, Cephalota (Taenidia) dulcinea López, Rosa & Baena, 2006, Cicindela (Cicindela) campestris campestris Linnaeus, 1758, Cicindela (Cicindela) maroccana Fabricius, 1801, Cylindera (Cylindera) paludosa (Dufour, 1820), Lophyra (Lophyra) flexuosa flexuosa (Fabricius, 1787), and Myriochila (Myriochila) melancholica melancholica (Fabricius, 1798). This assemblage of tiger beetles in La Mancha represents 41% of the overall diversity of tiger beetles present in the Iberian Peninsula (based on Serrano 2013). Taxonomic assignation at subspecific level for La Mancha populations of *Cicindela maroccana* is problematic based on the scarce material studied. Nomenclature used follows Putchkov and Matalin (2003) and Serrano (2003, 2013).

Observations on local geographic distribution, general landscape selection (soil structure and vegetation types) and adult activity are described in the following paragraphs, sorted by species.

**Table 3.** Number of specimens of tiger beetles found at each sampled locality.

No.	Lake name / Species	*Calomera littoralis*	*Cephalota maura*	*Cephalota circumdata*	*Cephalota dulcinea*	*Cicindela campestris*	*Cicindela maroccana*	*Cylindera paludosa*	*Lophyra flexuosa*	*Myriochila melancholica*
1	Laguna de El Longar	133	8	3	39	9		8		6
2	Laguna del Altillo Chico	2		43	12			16		
3	Laguna del Altillo Grande			11	27			16		
4	Laguna de la Albardiosa									
5	Laguna Larga	49						1		
6	Laguna de Tirez	2		21	35	9	4			4
7	Laguna de Peña Hueca	15	1	4	30		2			
8	Laguna del Taray				11			1		
9	Laguna Grande	22								
10	Laguna Chica									
11	Laguna Grande	68	8		1					
12	Laguna de la Sal	2			6		2			
13	Laguna de los Carros				68					
14	Laguna de Pajares		10		40			3		
15	Laguna de las Yeguas	22	1	6	66	1		8		
16	Laguna Camino de Villafranca	48	13		2					11
17	Laguna de la Veguilla									
18	Laguna de Salicor	11		1		10				
19	Laguna de Retamar									
20	Laguna del Pueblo	51								14
21	Laguna de Navalafuente					1				
22	Laguna de Alcahozo	4		12	10					
23	Laguna de Manjavacas	25	20							
24	Laguna de Sánchez Gómez	4			95	1				
25	Laguna de la Dehesilla	52			30	12		2		
26	Laguna de Alcahozo Chico	42				28		4	3	
27	Laguna del Taray Chico					3				
28	Laguna de El Hito				29					
29	Cerro San Cristóbal				23					
30	Arroyo de San Marcos		3			4		1		
31	La Laguna de Miguel Esteban	9	30							
32	Gigüela marshes							7		

### *Calomera littoralis littoralis*

**Figure 4.**
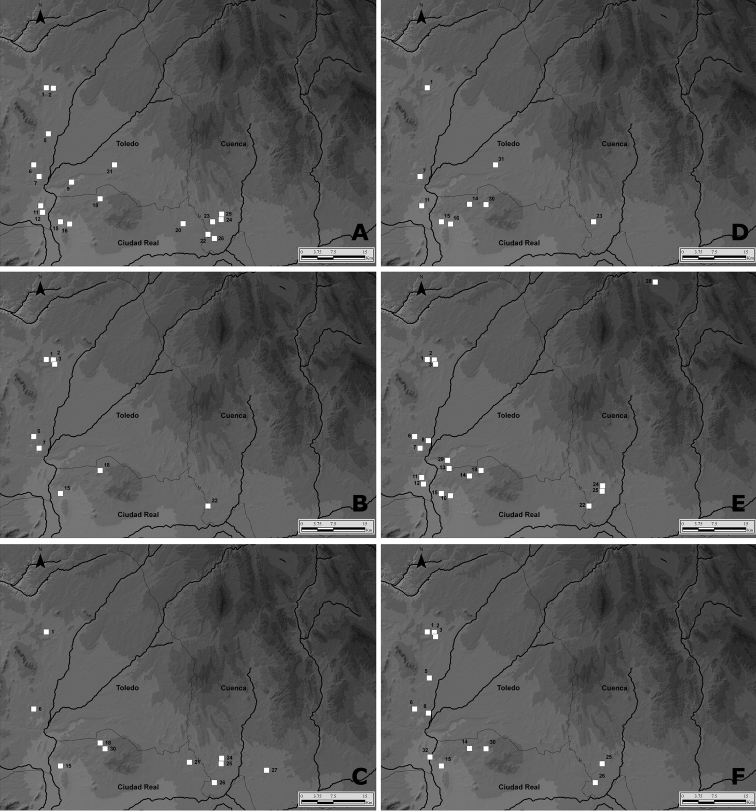
Maps showing the location of species of Cicindelinae found in the wetlands of La Mancha. Numbers correspond to Fig. 1. **A** *Calomera littoralis littoralis* **B** Cephalota (Cassolaia) maura **C** Cephalota (Taenidia) circumdata imperialis **D** Cephalota (Taenidia) dulcinea **E** *Cicindela campestris campestris* **F** *Cylindera paludosa*.

**Figure 5.**
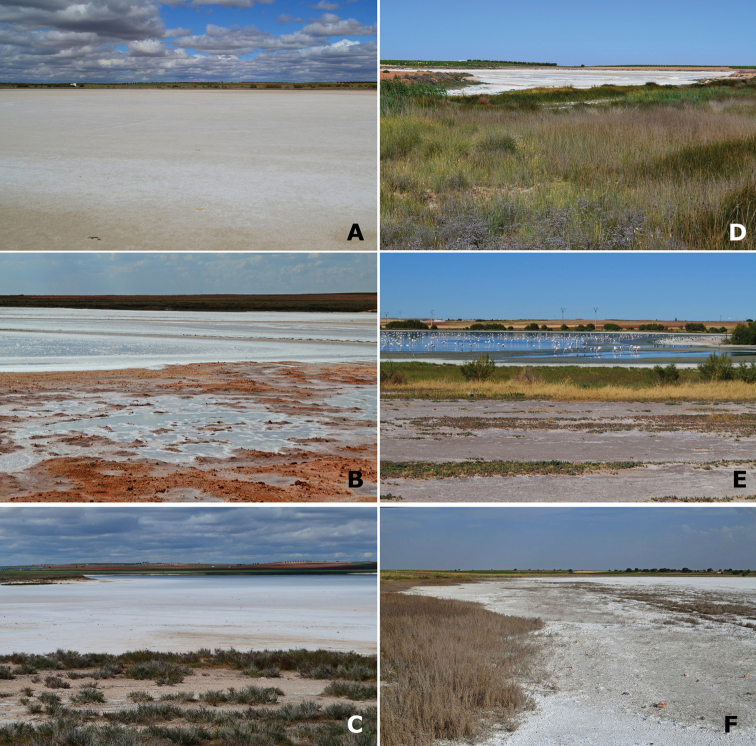
Representative habitats of Cicindelinae in La Mancha. **A** Laguna de la Sal (Toledo) in May. Salt flats occupied by *Calomera littoralis littoralis* **B** Laguna de Peña Hueca (Toledo) in April. Wet salt flats, with gypsum outcrops, occupied by *Calomera littoralis littoralis*; when totally dry, in summer, occupied by *Cephalota circumdata imperialis* **C** Laguna de Las Yeguas (Ciudad Real) in June. Salt flats, occupied by *Calomera littoralis littoralis* and *Cephalota circumdata imperialis*; and halophytic prairies occupied by *Cephalota dulcinea* and *Cylindera paludosa* **D** La Laguna de Miguel Esteban (Toledo) in July. Salt flats, occupied by *Calomera littoralis littoralis*; and halophytic prairies with “albardinal” occupied by *Cephalota maura maura* **E** Laguna del Camino de Villafranca (Ciudad Real), with flamingos, in July. Sapropel shores occupied by *Calomera littoralis littoralis* **F** Laguna de Alcahozo (Ciudad Real) in June. Salt flats, occupied by *Calomera littoralis littoralis* and *Cephalota circumdata imperialis*; and open areas near dry halophytic prairies occupied by *Cephalota dulcinea* and also by *Cephalota circumdata imperialis*. Photographs by N. Percino and MG-P.

**Figure 6.**
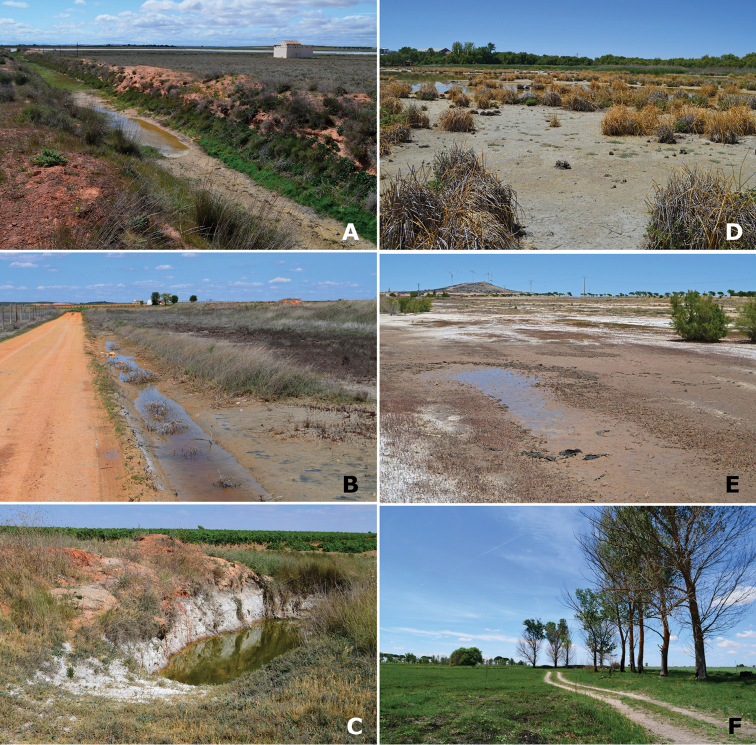
Representative habitats of Cicindelinae in La Mancha. **A** Channel at Laguna de las Yeguas (Ciudad Real) in May. Wet areas occupied by *Calomera littoralis littoralis* and *Cicindela campestris*; when drier, occupied by *Cephalota maura maura* **B** Road side pools and halophytic vegetation in Laguna de Manjavacas (Cuenca) in April. *Calomera littoralis littoralis* was the only species observed **C** Wells and deep pools in Laguna de Manjavacas (Cuenca) in June. *Cephalota maura maura* is frequent in these structures **D** Laguna del Pueblo de Pedro Muñoz (Ciudad Real) in July. Mud areas amongst hydrophytic matts, occupied by *Calomera littoralis littoralis* and *Myriochila melancholica melancholica* **E** Temporary pools at Laguna del Longar (Toledo), in July. *Calomera littoralis littoralis*, *Myriochila melancholica melancholica* and *Cephalota maura* co-occcur at the sides **F** Laguna del Taray Chico (Cuenca) in May. Trails and non-halophytic prairies near the fresh water lake, occupied by *Cicindela campestris campestris*. Photographs by N. Percino and MG-P.

*Calomera littoralis littoralis* (Fig. 2a) was found in 19 localities (Fig. 4a). A total of 561 specimens were observed. Most specimens were found in relatively humid situations: in the humid shore of small lakes (45.6%), around drying pools (25.7%), in drying channels communicating saline lakes (3.6%), and a few in totally dried out lakes (25.1%). Most specimens were found in saline areas (66.8%) (Table 2) (Figs 5–6), but they were also present in ponds with much less salt concentration (33.2%), located near a salt lake (El Longar), or water bodies resulting from regulated flooding (south end of Laguna del Camino de Villafranca, and Laguna del Pueblo de Pedro Muñoz) (Fig. 6d, e).

**Figure 7.**
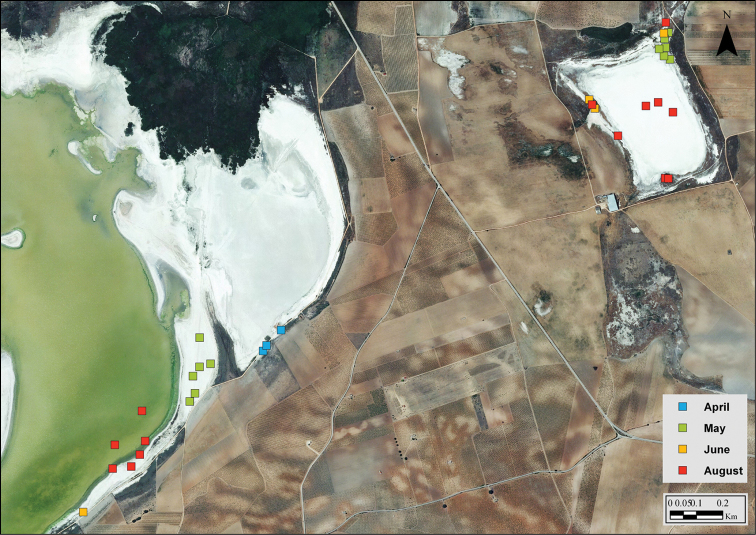
Observations of *Calomera littoralis littoralis* at Laguna de Manjavacas (left) and La Dehesilla (upper right). Colours indicate the month in which observations were made (see legend). Note seasonal changes in specimen’s location as the water front retreats or following changes in humidity of the soil. Blue squares correspond to the habitat shown in Fig. 6b.

Most populations of *Calomera littoralis* changed location within the same locality during the period of study. We followed these intra-populational movements at some lakes. At Laguna de Manjavacas, specimens were active in late April in the salty marshes surrounding the main, totally water- filled, salt lake. As the lake started to dry out, specimens appeared along the shoreline, and disappeared completely from the dried out salty marshes. Subsequently, and until the complete drying out of the main lake, specimens of *Calomera littoralis*, moved from nearby peripheral vegetated areas towards central flat drying areas (Fig. 7). In general terms, the spatial positioning of specimens of *Calomera littoralis* accompanied the retreat of water, following the wet soil. After total drying, some specimens remained active in local humid patches at different positions within the lakes, until their total disappearance.

Most specimens were located on salt crust (40.5%), in open denuded areas (48.8%) (Table 2). No specimens were found on roads, or in shallow marshes or channels located far from large lakes, but they were present around all large water bodies, including nearby freshwater reservoir shores (Carrascosa del Campo, Cuenca).

**Figure 8.**
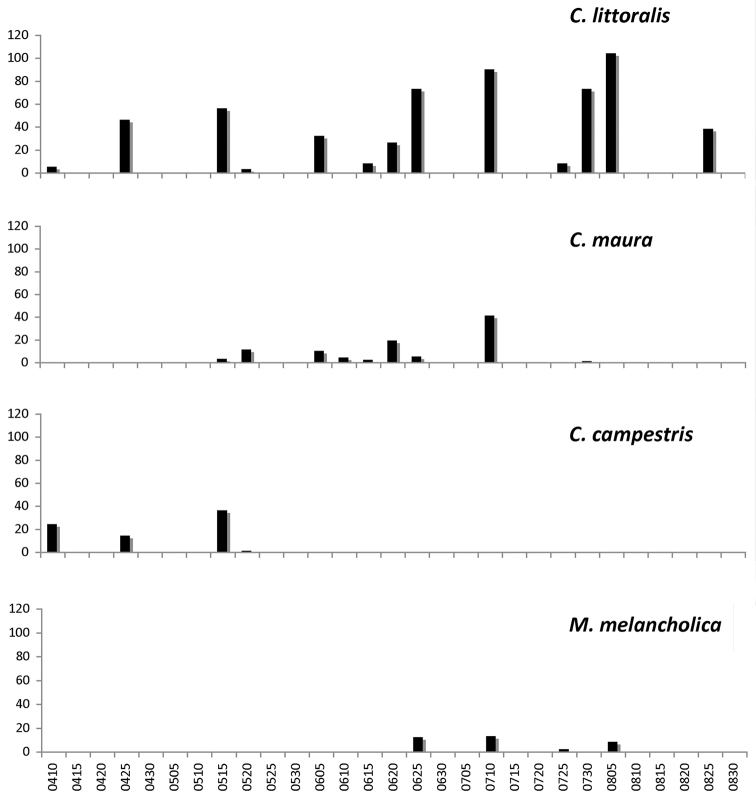
Frequency of observation of Cicindelinae during the sampling period (April to August), all sites pooled. From top to bottom: *Calomera littoralis littoralis*, *Cephalota maura maura*, *Cicindela campestris campestris*, and *Myriochila melancholica melancholica*.

Specimens of *Calomera littoralis* were present in the area all over the study period (Fig. 8), although active adults were almost never continuously present in the same location through the entire sampling, with marked local absences following the complete drying out of each lake. Specimens were observed active in February 2015 (Las Yeguas, Fig. 1: 15, P. Pichaco pers. com.). Our phenological observations are comparable with previous data for the region (Lencina et al. 1991), partially coincident with those from Tunisian populations (Jaskuła and Rewicz 2015), but quite different from published data from other geographic regions (Vives and Vives 1978b, Zanella 2010, both for *Calomera littoralis nemoralis* Olivier, 1790).

Abundance of *Calomera littoralis* is highly variable across the study area (Table 3; Suppl. material 1). Since specimens are generally present near water, some pools on the drying lakes might have extraordinary concentrations of specimens (more than 6 per square metre), while they are generally less concentrated when water is largely available, especially at lake shores. Aggregation of *Calomera littoralis* adult specimens has been correlated with habitat disturbance and prey availability (Mazzei et al. 2014); our data suggest that concentration seems to be a direct effect of availability of wet soils, rather than a response to food or disturbances.

*Calomera littoralis* was occasionally found in sympatry with *Cicindela campestris* in late spring (La Dehesilla, Camino de Villafranca Channel, Alcahozo Chica), with *Cicindela maroccana* (La Sal), and with *Cephalota circumdata* (Altillo Chica, Tirez, Peña Hueca), but strict syntopy, sharing a unique patch, was only observed with *Myriochila melancholica* at the end of summer (El Longar, Tirez, Camino Villafranca, and Pedro Muñoz), and occasionally with *Cephalota maura* (El Longar, Camino de Villafranca Channel) and with *Lophyra flexuosa* (Alcahozo Chica) in summer. When present, *Calomera littoralis* is generally the dominant species, except in extreme drying-out situations at the lake basins or channels, when *Cephalota circumdata* or *Cephalota maura* might be temporally dominant.

*Calomera littoralis* is a conspicuous and very active species, displaying its activity in plain sunlight. When approached, they run fast, and when disturbed they are able to start a quite long and sustained, relatively elevated, flight, sometimes changing direction in mid- flight. Flight is never directed to vegetated areas. At some localities (Manjavacas, Peñahueca, Alcahozo Chico) many specimens remain quiet among the sparse vegetation of the basin shore, running towards the central open areas when disturbed. One specimen was observed at night, hidden, but alert, in the crevices of a thick salt crust layer (Peña Hueca). Jaskuła et al. (2005) mentioned that some specimens climb small bushes for the night. Nocturnal activity may be extended but not common for this species, since attraction to artificial lights was reported for *Calomera littoralis nemoralis* in Northern Italy (Zanella 2010). Copulatory behaviour was observed during most of the study period. Predatory behaviour was observed towards ants and small flies. Some specimens captured their prey while running away from our chase.

### Cephalota (Cassolaia) maura maura

*Cephalota maura maura* (Fig. 3b) was found in 12 localities (Fig. 4b). The 96 specimens observed were found in relatively humid situations, including salt marshes (37.5%) (Laguna de Miguel Esteban; Peña Hueca), inclined saline sides of channels (7.3%) (Salicor; San Cristóbal; El Longar), drying channels communicating saline lakes (15.6%) (Camino de Villafranca), around drying pools and pits (24%) (Manjavacas, El Longar), but also in quite dry areas, such as dirt roads covered by a flat salt crust (15.6%) (Pajares trail) (Fig. 5d and 6a,c,e). All the specimens were found in saline areas (96.9%), but they were also present in less saline drying pools (El Longar) (Fig. 6e). They avoided the basins of large salt lakes, with most of the populations located in small saline areas separated from the main lake. Many populations, particularly the denser ones, were found in man-modified habitats, such as ancient ditches with salty sides, or the inclined banks of old pits excavated not far from the lakes. Márquez Rodríguez (2014) also reported the presence of the species in degraded habitats in southern Spain. Most places where they were present in La Mancha kept water through all the sampling period. Additional specimens were found under similar conditions in man-modified salt marshes not included in the regional study (Belinchón in Cuenca and Yepes in Toledo).

Most specimens were located on salt patches in trails (20.8%), and on banks and other compact soils (39.6%). According to Márquez Rodríguez (2014), *Cephalota maura* uses banks of streams for reproduction. Most specimens were found on bare ground (65.6%) and secondarily in halophytic prairies (27.1%) (Table 2). No specimens were found in the basins of large lakes.

Adult specimens of *Cephalota maura* were observed through most of the study period (Fig. 8). However, specimens of *Cephalota maura* were not continuously present in the same location during the entire period. Absences did not follow a clear pattern as in *Calomera littoralis*, since populations disappeared even if humidity was still present. Population densities of *Cephalota maura* are variable, but generally low in comparison to *Calomera littoralis*. It is not a rare species, but its presence in non-conventional habitats requires further exploration. Besides, some populations occupy habitats of minimal extension (saline sides of isolated pits in halophytic prairies), making their localization difficult.

*Cephalota maura maura* was occasionally found in strict syntopy with *Cicindela campestris* in late spring (El Longar, Salicor and San Cristóbal channels), with *Cephalota dulcinea* and *Cylindera paludosa* (road to Pajares) in early summer, with *Calomera littoralis* in mid and late summer (Laguna Grande de Villafranca, El Longar, Camino Villafranca channel), and with *Myriochila melancholica* at the end of summer (El Longar).

Specimens of *Cephalota maura maura* are very active, displaying most of its activity in plain sunlight, although some specimens were observed resting on the ground between the twigs of *Scirpus* at mid-day, near the water (La Laguna de Miguel Esteban, Laguna Grande de Villafranca). When approached, they run fast, displaying a short low flight when threatened, often directed towards vegetated areas, or even into the water surface, from where they reach the shore.

### Cephalota (Taenidia) circumdata imperialis

*Cephalota circumdata imperialis* (Fig. 3c) was found in 8 localities (Fig. 4c). The 101 specimens observed were found inside the saline basin of small lakes, distributed both near the shore, or in central areas (Fig. 5b, c, f). No specimens were found outside the salt flats. Specimens were found mainly on salt crusts (81.2%) and in sun exposed granulated salty soils (82.2%) (Table 2).

**Figure 9.**
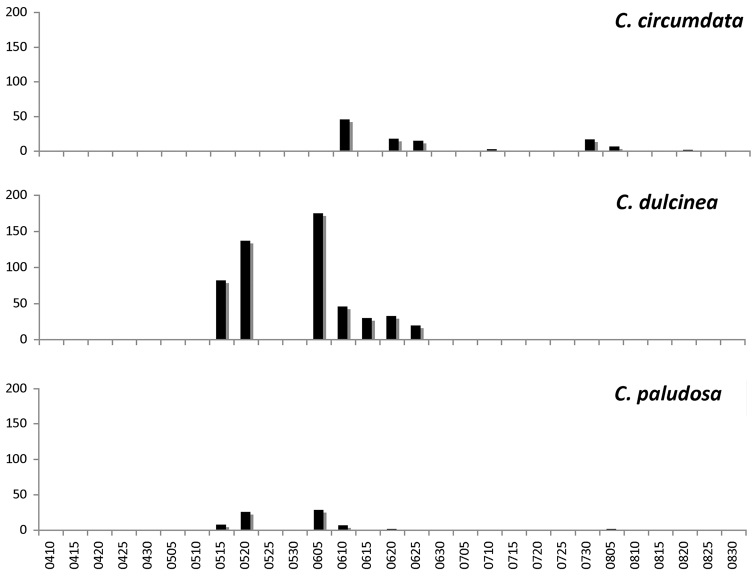
Frequency of observation of Cicindelinae during the sampling period (April to August), all sites pooled. From top to bottom: *Cephalota circumdata imperialis*, *Cephalota dulcinea*, and *Cylindera paludosa*.

Specimens of *Cephalota circumdata* were present in the area during summer, from late June to August (Fig. 9), as previously reported (Vives and Vives 1978b, Lencina et al. 1991). Active adults were almost never continuously present in the same location throughout the entire period, with marked local absences following the complete drying of the mud beneath the salt layer.

Population densities of *Cephalota circumdata* are variable, but normally low. Single specimens were often found alone, far from any other specimen, and when close, they were usually relatively isolated, and well distributed along favourable habitat. *Cephalota circumdata* was occasionally found in sympatry with *Calomera littoralis* (Altillo Chica, Tirez, Peña Hueca, Salicor), and with *Cephalota dulcinea* at the start of summer (El Longar, Las Yeguas, Alcahozo).

**Figure 10.**
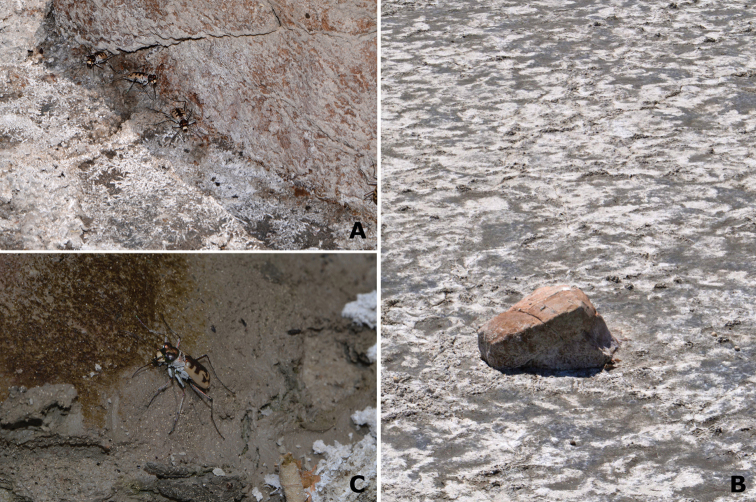
Behavioural observations on *Cephalota circumdata imperialis*. **A, B** A few specimens of *Cephalota circumdata* located at the shade of a rock in the middle of the exposed salt flat **C** Individual photographed on the wet mud under the remains of a plastic bag, also in the salt flat. This behaviour is atypical for the species, which is generally active at full sunlight in mid summer in the area. The observations were made the 13th of June, at mid-day, the first date in which activity of the species was recorded. It is possibly that the specimens had just eclosed and wait in the shade while hardening their integument. Photographs by MG-P.

Most specimens were observed displaying their activity in plain sunlight, in agreement with previous observations of circadian activity of *Cephalota circumdata leonschaeferi* in Italy (Eusebi et al. 1989). They run fast when approached, or fly relatively far away towards the centre of the lake. Flight was not directed to vegetated areas. However, most specimens started running from relatively hidden positions, either from below dry twigs, or from under isolated plant mats. The earliest observations of the species in late June, were made on Altillo Grande and Chico lakes. All the specimens observed at that time were located under a few random objects (stones, bricks, wood, plastic bags) present in the salt flats. The specimens were, either aggregated under the shade produced by the objects (Fig. 10a), or aggregated under the objects, in direct contact with the wet mud (Fig. 10b). Further searches during the species activity period failed to disclose any other specimens hidden or aggregated under objects. Lencina et al. (1991) indicated that specimens of *Cephalota circumdata* aggregate under large stones at dusk.

### Cephalota (Taenidia) dulcinea

**Figure 11.**
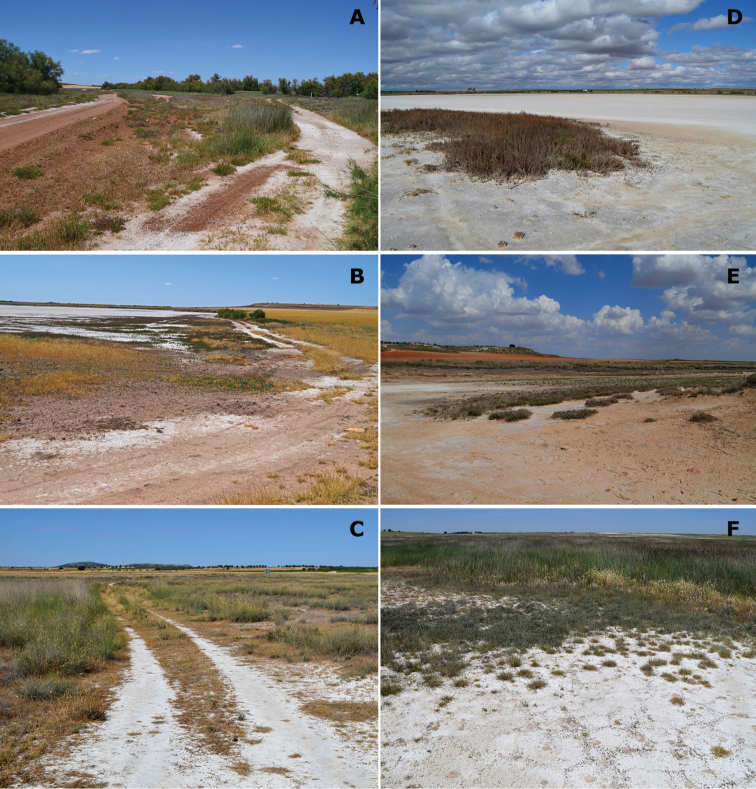
Characteristic habitat of the La Mancha endemic tiger-beetle Cephalota (Taenidia) dulcinea. **A** Trails at laguna del Taray (Toledo) **B** Trails and halophytic vegetation in Laguna de Pajares (Ciudad Real), where it was found in company of *Cephalota maura maura* **C** Trails and “albardinal” in Laguna del Altillo Chica (Toledo), where it co–occurs with *Cylindera paludosa* **D** Open areas amongst halophytic vegetation at laguna de La Sal (Toledo), *Calomera littoralis littoralis* and *Cicindela maroccana* occur in this habitat at the end of the spring **E** Halophytic vegetation at Laguna de Peña Hueca (Toledo), in this spot, *Calomera littoralis littoralis* and *Cicindela maroccana* are present at the end of the spring **F** Halophytic vegetation and open areas with salty granulated soils at Laguna de Sánchez Gómez (Cuenca). Photographs MG-P.

*Cephalota dulcinea* (Fig. 3a) was found in 18 localities (Fig. 4d). The 524 specimens observed were found in relatively dry situations. Most of the specimens were found on small open ground areas, generally covered by a loose, granulated, sometimes dusty soil layer (53.2%), which gets a little more compact after typical summer storms. Other specimens were located on dirt roads and trails (45.8%), some of them covered by salt patches (29.2%). All of the specimens were found in saline areas, either lake shores and surrounding trails, or salt marshes (Figs 5c, f and 11a–f). Vegetation cover was never dense, but almost consistently present, except along trails; 55.2% of the specimens were located around typical halophytic vegetation, 23.3% on open *albardinal*, and 21.6% in open denuded areas.

Active adults were observed from late spring to midsummer (Fig. 9), although they were almost never continuously present in the same location during the entire period, disappearing completely or changing locations within the same locality. The latest observations correspond to slow moving specimens, missing tarsal segments, likely corresponding to the actual end of the activity period.

Changes in population extension and local positioning were marked in some lakes, particularly at Los Carros, Sánchez Gómez and La Dehesilla, while in other areas the specimens occupy constantly at least a portion of the locality (Tirez, Peña Hueca, Las Yeguas). At Los Carros, La Dehesilla and Las Yeguas, large concentrations of specimens were present along dirt roads and trails in late spring (Fig. 11e, f). These trails were totally abandoned by midsummer, and the remaining active specimens were located in open small salt flats, in vegetated dry areas. Frequency of observation of *Cephalota dulcinea* is variable, but in general densities are relatively high, especially in late spring and early summer, and the species is generally easy to detect (Table 3). We failed to find the species at Salicor, where one specimen was found in 2012.

*Cephalota dulcinea* was occasionally found in sympatry with *Cephalota circumdata*, but strict syntopy, sharing a unique patch, was only observed with *Cylindera paludosa* at the end of spring in open flats within vegetated areas, and occasionally with *Cephalota maura* along trails. When present, *Cephalota dulcinea* is generally the dominant species, and it was the only species found in some small lakes (Los Carros).

**Figure 12.**
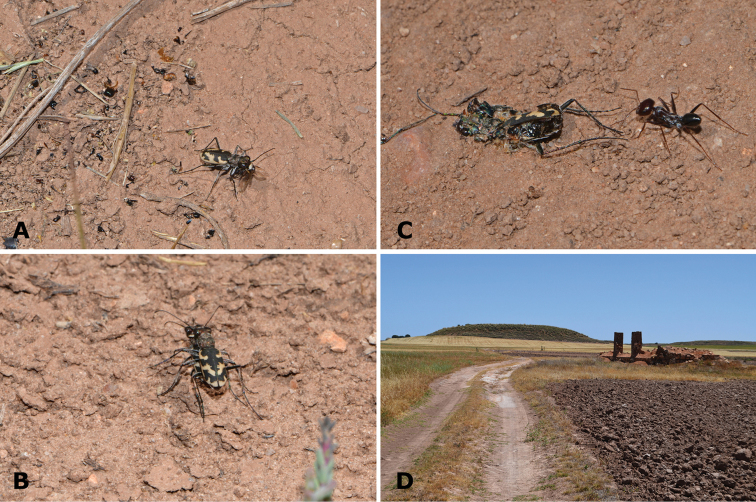
Behavioural observations on *Cephalota dulcinea* **A** Specimen of *Cephalota dulcinea* located in a trail devouring a winged hymenopteran. Note the number of insect remains located behind the specimen (ants, other Hymenoptera and Coleoptera), suggesting that the spot is used as a hunting post **B** A couple of *Cephalota dulcinea* involved in courship **C** A recently killed specimen of *Cephalota dulcinea* in a trail, about to be seized by ants **D** Trail located near laguna de Pajares with a large colony of *Cephalota dulcinea*, *Cephalota maura maura* and scattered *Cylindera paludosa*. We hypothesize that these trails, despite of being surrounded by unfavourable habitat, might be used as dispersal corridors between lakes, facilitating the maintenance of metapopulation systems. Photographs by MG-P.

*Cephalota dulcinea* is an active species, displaying its activity in plain sunlight. When approached, they run fast and fly relatively short distances, sometimes changing direction in mid- flight. Flight can be directed to vegetated areas, but usually they fly farther along trails or saline flats. Occasionally, we found some specimens resting in the shade of objects, at midday (El Longar). Preying behaviour was observed towards ants, other small hymenopterans, and small dipterans. Some specimens seem to keep a hunting spot for a period, as suggested by the insect remains scattered around them (Fig. 12a). Copulatory behaviour was observed during most of the study period (Fig. 12b).

### Cicindela (Cicindela) campestris campestris

*Cicindela campestris campestris* (Fig. 2b) was found at 11 localities (Fig. 4e). All 78 specimens found were present in relatively humid situations, most of them in the granulated salty soil areas around small lakes (57.7%), some in drying channels communicating saline lakes (16.7%), or in small open areas (15.4%), and roads near prairies or cultivated fields (9.0%). Specimens were found around saline or freshwater areas, in densely vegetated areas (42.3%), or relatively open (26.9%) (Table 2) (Fig. 6a, f).

Specimens of *Cicindela campestris* were present in the area in late spring (Fig. 8). According to previous studies, the species is also present during fall and in early spring in Castilla–La Mancha (Lencina et al. 1991, 2001), consequently in this study we missed a large part of its possible activity period. Three additional specimens were observed in October at Tirez, outside the monitoring period.

Population abundances of *Cicindela campestris* are highly variable (Table 3). They can be locally abundant (La Dehesilla, Salicor, Alcahozo Chica), or represented by isolated specimens, sometimes found in the middle of other species populations (*Cephalota maura*). *Cicindela campestris* was occasionally found in strict sympatry with *Calomera littoralis* in late spring (La Dehesilla, Camino de Villafranca Channel, Alcahozo Chica), with *Cephalota maura* in drying channels (El Longar, Camino de Villafranca Channel), and with *Lophyra flexuosa* (Alcahozo Chica) in early summer. It is the only species found in some largely vegetated less saline or non-saline areas (Taray Chico, Navalafuente).

*Cicindela campestris* is an active but not conspicuous species in the area, displaying its activity in small patches and trails amongst vegetation. When approached, they run along the trails or into vegetation, and when flying, they show a tendency to land on vegetation. Copulatory behaviour was observed during most of the study period.

### Cicindela (Cicindela) maroccana

*Cicindela maroccana* (Fig. 2c) was only found in three localities (Fig. 1: 6, 7, 12). Only 8 specimens were found, all of them located in open or poorly vegetated areas of the saline shore of small lakes and salty marshes, on granulated soils (Fig. 11d, e).

Specimens of *Cicindela maroccana* were present in the area during late spring. According to previous studies, the species is also present in early spring (Lencina et al. 1991, Ortuño and Toribio 1996). These reports are now confirmed for the area since the species was observed active in February 2015 (Las Yeguas, Fig. 1: 15, P. Pichaco pers. com.). Frequency of observation of *Cicindela maroccana* is low in the study area, but the species requires an earlier sampling (Table 3). *Cicindela maroccana* was found in sympatry with *Cicindela campestris* (Tirez), and with *Calomera littoralis* (La Sal, Peña Hueca). The specimens found displayed their activity in plain sunlight, flying readily when disturbed.

### Cylindera (Cylindera) paludosa

*Cylindera paludosa* (Fig. 2d) was found in 11 localities (Fig. 4f). 67 specimens were found in areas with a relatively dense vegetation cover, most of them in saline flats in the *albardinal* (*Lygeum spartum* prairies) (38.8%), in open halophytic prairies (41.8%), or in dense halophytic prairies (11.9%) at the shore of small lakes (Table 2) (Figs 5c and 12c).

Specimens of *Cylindera paludosa* were present from late spring to midsummer (Fig. 9), although one isolated specimen was found at the end of August. Population densities of *Cylindera paludosa* are variable. The species may be locally abundant in salt flats around or inside the *albardinal* (Table 3).

*Cylindera paludosa* was occasionally found in sympatry with *Cephalota dulcinea*, around salt flats and vegetated saline marshes (Altillo Chica, El Longar, Las Yeguas, La Dehesilla), with *Cicindela campestris* in late spring (Salicor), and with *Cephalota maura* (El Longar channel, Pajares trail). *Cylindera paludosa* is generally the dominant species in salt marshes, and often it is the only species present in marshes of limited extension. *Cylindera paludosa* is an inconspicuous but very active species, displaying its activity in plain sunlight. When approached, they fly readily towards the nearby vegetated areas, rendering its observation difficult.

### Lophyra (Lophyra) flexuosa flexuosa

*Lophyra flexuosa flexuosa* (Fig. 2e) was only found at one locality (Alcahozo Chico, Fig. 1: 26) (n=3) during the study period, but it was observed in another locality (Salicor, Fig. 1: 18) (n=5) during the preliminary samplings in 2012. The specimens were found in the saline shore of a small lake (2012), or in a drying- out salt marsh (2014). We failed to find the species at Salicor in 2014, despite successive searches.

We found the specimens in late spring (mid May 2014, and early June 2012). Despite successive samplings, the observations were made in a single occasion. These observations are puzzling, because, according to the literature and personal observations, the species has a relatively long period of activity, from April to July, in other areas of Castilla – La Mancha and nearby Madrid (Lencina et al. 1991, Ortuño and Toribio 1996, unpub. observ.). The population of *Lophyra flexuosa* at Alcahozo Chico was found in strict sintopy with *Calomera littoralis*, and very close to *Cicindela campestris* and *Cylindera paludosa*. At Salicor it was the only species present on the shore. *Lophyra flexuosa* is a very active species, displaying its activity in plain sunlight, and flying readily when disturbed.

### Myriochila (Myriochila) melancholica melancholica

35 specimens of *Myriochila melancholica melancholica* (Fig. 2f) were found in 4 localities (Fig. 1: 1, 6, 16, 20). All specimens were found in relatively humid situations, all of them on the shore of small lakes or around drying pools. Some of the specimens were found in saline areas (Laguna de Tirez), but most of them were present in drying pools, with much less salt concentration, located near a salt lake (El Longar), or lakes resulting from regulated flooding (south end of Laguna del Camino de Villafranca, and Laguna del Pueblo de Pedro Muñoz) (Fig. 6d, e). Most of the specimens were observed on mud amongst open hydrophytic vegetation (71.4%), or in denuded shores (28.6%) (Table 2).

The period of activity of *Myriochila melancholica* was restricted to the end of the study period, from late June to early August (Fig. 8). The population of Laguna del Pueblo de Pedro Muñoz moved along the shoreline, following the retreating water front, as the small lake was drying out once the forced flooding was stopped. Populations disappeared following the complete drying- out of the pools (Tirez, El Longar).

Frequency of observation of *Myriochila melancholica* in the area is low, with only a few specimens found per visit. *Myriochila melancholica* was always and only found in syntopy with *Calomera littoralis*. In these cases *Calomera littoralis* was the dominant species. *Myriochila melancholica* displayed its activity in plain sunlight, but it is also attracted to artificial lights (Ortuño and Toribio 2002). When approached, they usually run fast towards wetter areas, but generally stop at a relatively short distance from the starting point. They only fly when distressed.

## Discussion

### Tiger beetle assemblage at La Mancha wetlands

The tiger beetle taxocenosis in La Mancha wetlands is composed of 9 species (Table 3; Figs 2–3). This is a high number of cicindelids for any small geographic area in Europe. In fact, Jaskuła (2011) recorded a maximum of 8 species per square of 1º latitude and longitude in the Balkan Peninsula. This region, together with the Iberian Peninsula, displays the largest diversity of tiger beetles in Europe. The studied region in La Mancha fits entirely inside a single 1º latitude/longitude square (longitude west 2º40’at Laguna del Hito, to 3º22’ at Laguna de Tirez; and latitude north 39º21’ at Laguna de Alcahozo Chico, to 39º53’ at Laguna del Hito) (Fig. 1). All the entire tiger beetle community is actually represented in a surface of less than 2 km^2^. According to these data, La Mancha is likely the European geographic area with the most complex assemblage of tiger beetles (see Cassola 1970, 1972, Aliquò 1992, Franzen 2005, Jaskuła et al. 2005, Jaskuła 2007, 2011, Kirichenko et al. 2001).

The cicindelid assemblage at La Mancha wetlands could be even more complex, since two additional species of tiger beetles, not found in the study area, inhabit the region of Castilla – La Mancha. *Cicindela lagunensis* has been located in sandy soil areas, relatively close to La Mancha wetlands (Ortuño and Toribio 1996, Matalin 1998, Cardoso and Vogler 2005). We have not located the species in the study area, but we cannot exclude the possibility that it could be present along the banks of the rivers (Riansares and Gigüela) that cross the region. *Grammognatha euphratica euphratica* (Dejean, 1822), has been recorded at the extreme south-eastern area of Castilla – La Mancha, about 150 km south east of the study area (Ortiz et al. 1988, Andújar et al. 2002). Although *Grammognatha euphratica* inhabits salt marshes, its ecological characteristics seem to differ from those available at La Mancha wetlands. However, in current scenarios of climate change, the possibility of colonization of La Mancha salt marshes cannot be excluded.

Such a concentration of cicindelid beetles, 9 species in an area of less than 1º latitude/longitude square, is likely the result of a combination of both historical (paleogeographic) and ecological factors. The presence of one endemic species, *Cephalota dulcinea*, suggests that, on the one hand, the region has maintained a relative level of historical isolation with respect to other Iberian areas. On the other hand, some areas acting as sanctuaries of diversity (*sensu* Recuero and García-París 2011), might have persisted in the high plateau of La Mancha, despite the strong changes suffered during the Pleistocene glacial periods (Zagwijn 1996). It seems possible that these sanctuaries were precisely the saline lakes, which currently harbour a relative high level of endemic and relict species (e.g. carabid beetles as *Broscus uhagoni* Bolívar, 1912; bradyporine orthopterans as *Pycnogaster graellsii* Bolívar, 1873) (Gómez et al. 1991, Toribio and Pichaco 2014).

The diversity of environments available for tiger beetles at La Mancha wetlands, also contributes to the maintenance of such a high number of cicindelids, since most of the species seem to occupy particular ecological niches within the area (Fig. 14, Table 2). Spatial segregation of tiger beetles based on habitat structure has been reported in previous studies (Willis 1967, Cassola 1970, Ganeshaiah and Belavadi 1986, Ortíz et al. 1988, Schultz 1989, Lencina et al. 1991, Hoback et al. 2000, Satoh and Hori 2005, Jaskuła 2011, 2015), suggesting that it is a common situation for the group.

**Figure 13.**
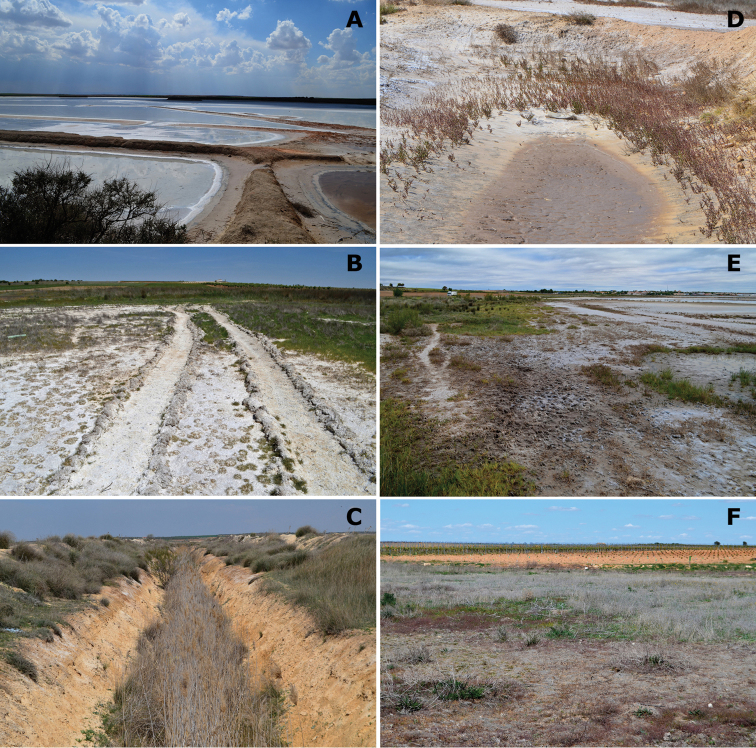
Conservation problems for tiger beetles in La Mancha, and remains of old semi-industrial activities which provide additional structural heterogenity **A** Saline pools at Laguna de Peña Hueca (Toledo) occupied at different seasons by *Calomera littoralis littoralis*, *Cephalota dulcinea*, *Cephalota circumdata imperialis* and *Cephalota maura maura* **B** Trails on salty soils at Laguna de la Dehesilla (Cuenca), where *Calomera littoralis littoralis*, *Cicindela campestris campestris* and *Cephalota dulcinea* are present along different periods of the year **C** Old channel in Laguna del Longar (Toledo), where *Cephalota maura maura*, *Cicindela campestris* and *Cylindera paludosa* co-occur **D** Saline pool at Laguna de Tirez (Toledo), where *Calomera littoralis littoralis* and *Myriochila melancholica melancholica* co-occur, while populations of *Cephalota dulcinea* and *Cephalota circumdata imperialis* are established not far from this point **E** Effect of sheep along the shores of the saline Laguna Grande de Quero (Toledo), in this spot *Calomera littoralis littoralis* was present **F** Vineyards at the edge of Laguna de Alcahozo Chico (Cuenca), note the diverse halophytic vegetation and nearby open areas where *Calomera littoralis littoralis*, *Cicindela campestris campestris*, *Cylindera paludosa*, and *Lophyra flexuosa flexuosa* live in close proximity. Photographs by N. Percino and MG-P.

**Figure 14.**
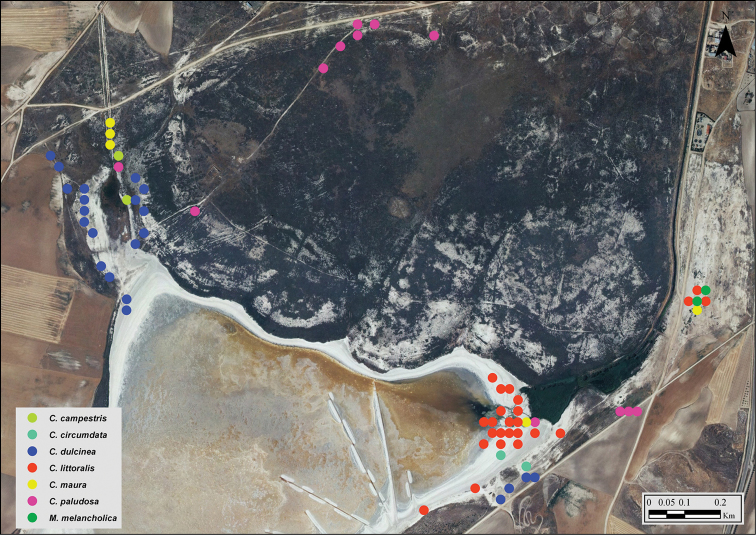
Location of seven species of Cicindelinae at Laguna del Longar (Lillo, Toledo). Colour dots indicate the spatial position of each specimen (often, more than one specimen are presented under a single dot) (see legend). Note the spatial segregation of most species. Dots in lower right area, where *Myriochila melancholica melancholica* was found, correspond to the habitat shown in Fig. 6e. Dots in the upper left area, where *Cicindela campestris campestris* was present, correspond to the habitat shown in Fig. 13c.

Habitat partitioning among species in La Mancha is evident at temporal and spatial scales. On the temporal scale, peaks of activity differ markedly among species (Figs 8–9). Only one species, *Calomera littoralis*, was present throughout the entire period of study. A set of two species, *Cicindela campestris* and *Cicindela maroccana*, were exclusively present during spring, and fall in the case of *Cicindela campestris* (although, as mentioned above, their period of activity likely started before our earliest sampling date), sharing a minimum overlap, with the second set. The second set of taxa, composed by *Cephalota maura*, *Cephalota dulcinea*, *Cylindera paludosa*, and *Lophyra flexuosa*, started their activity in late spring, overlapping occasionally with isolated late specimens of *Cicindela campestris*. *Cephalota dulcinea*, *Cylindera paludosa*, and possibly *Lophyra flexuosa*, ended their activity quite abruptly, during mid-late June, with isolated individuals of *Cylindera paludosa* observed at later dates. *Cephalota maura* activity overlapped with a third set of species, composed of *Cephalota circumdata* and *Myriochila melancholica*. This third group of taxa started their adult activity at mid and late June respectively, and were observed until late and early August (Figs 8–9). These observations are similar to those reported by Lencina et al. (1991) in a saline area in southeastern Castilla – La Mancha (Salinas de Pinilla).

*Cephalota dulcinea*, *Cephalota circumdata*, *Myriochila melancholica*, and possibly *Cicindela maroccana*, concentrate adult activity in a short period of time, in a single adult emergence period (Figs 8–9). *Cephalota maura*, *Cicindela campestris*, and possibly *Cylindera paludosa*, seem to present an extended period of emergence of adults, in two distinct periods (*Cicindela campestris* and possibly *Cylindera paludosa*), or in a temporally, but not geographic, continued single period (*Cephalota maura*). Emergence in *Calomera littoralis* seems to follow the same pattern as in *Cephalota maura*: temporally defined emergence at single localities, but temporally continuous in the whole geographic area (Figs 8–9).

Overlap in habitat use is maximal for *Myriochila melancholica* and *Calomera littoralis* when *Myriochila melancholica* is present, as previously reported by Lencina et al. (1991) in salt lakes from Albacete (Laguna de Pinilla) and Jaskuła et al. (2012) in Albania. *Myriochila melancholica* coexists in time and space with *Calomera littoralis*, and share the wet shores of drying pools and small lakes. We did not observe any kind of interaction between these two species, even though specimens of each species were found less than 30 cm apart from each other. Marked differences in size between *Myriochila melancholica* and *Calomera littoralis* may facilitate resource partitioning between them (Schoener 1974).

All other species show little overlap in habitat use (see Fig. 14 as a particular example where 7 species co-occur), as is typical for cicindelids all over the world (Freitag 1979, Knisley and Pearson 1984, Ganeshaiah and Belavadi 1986, Acciavatti and Pearson 1989). *Cephalota dulcinea* and *Cylindera paludosa*, are often present in the salt flats and trails within the *albardinal* (*Lygeum spartum* prairies), but *Cephalota dulcinea* is usually found in the less vegetated patches, where open spaces dominate, while *Cylindera paludosa* is usually located in narrower and smaller clearings, in denser patches (Table 2). *Cicindela campestris* and *Calomera littoralis* also occasionally share the humid margins of lakes, but in these situations *Calomera littoralis* occupies the open areas while *Cicindela campestris* is active around the vegetated patches. Frequency of syntopy is very low for all other species, usually represented by single observations, or by very low number of specimens.

Populations of some species remained on the same spot throughout their whole period of activity, from the earliest observations to the latest. Amongst those, *Cephalota circumdata*, *Cylindera paludosa* and *Cephalota maura* are the most representative, but also some populations of *Cephalota dulcinea* remained at the same location during their whole active period. Populations of *Calomera littoralis* and some of *Cephalota dulcinea*, moved to different patches within the same location throughout their period of activity. Populations of *Calomera littoralis* shifted their location following the retreat of water during the continuous drying-out of lakes and pools (Fig. 7). Location- shifting is a continuous process, in which the already active adult specimens and the newly emerged ones from successive cohorts are likely involved. Since we did not mark each individual, we cannot ascertain the contribution of each population group to the actual shifting. When the water surface of the lake dries out, there is a new shift in location; the beetles move to wetter patches in the salt basin, or disappear completely. We have not been able to determine if disappearances are a consequence of specimens’ deaths or migration to nearby lakes.

Apparent location shifts of *Cephalota dulcinea* were observed in dry lakes surrounded by trails and dirt roads, particularly at Las Yeguas and Los Carros, but also in other lakes on a smaller scale. Soon after the start of their activity, we observed large concentrations of specimens of *Cephalota dulcinea* along trails and roads around salt lakes, and less numerous at the salt basin shore in the typical halophytic prairies used by the species throughout the area. The number of specimens located on roads and trails declined as the season advanced, and completely disappeared well before the end of the general activity period of the species (Fig. 9). At the time of their disappearance, a reduced, but significant presence of specimens was located at the halophytic prairies, where they remained until the end of the activity period. Trails and roads are contiguous to prairies, so movements between them are likely to occur, but concentration of specimens at the prairies did not suffer dramatic changes, so it is possible that specimens occupying the roads die or move away, rather than move on to the prairies. A concentration of *Cephalota dulcinea* was found on a dirt road located between Laguna de Pajares, and Laguna de Salicor, about 600 m (air distance) from Pajares (Fig. 12d). This population might represent a migratory movement from Pajares, since we were only able to detect a couple of specimens of *Cephalota dulcinea* at the shore of the lake (Fig. 11b), and the road was totally flanked by unfavourable cultivated or ploughed fields (Fig. 12d).

These observations, coupled with the absence of *Cephalota dulcinea* at Laguna de Salicor, where it was found in 2012, suggest that, as a working hypothesis, the whole *Cephalota dulcinea* deme might function as a metapopulational system, with high probabilities of local extinction matched by frequent recolonization events (Diogo et al. 1999, Omland 2002, Cornelisse et al. 2013).

### Local threats for tiger beetle conservation at La Mancha wetlands

Populations of tiger beetles in La Mancha seems to present a relatively healthy status. Population densities are locally high for *Calomera littoralis littoralis*, *Cephalota maura*, *Cephalota dulcinea*, *Cylindera paludosa*, and *Cicindela campestris campestris*, a set of species with a relatively extensive area of occupation at La Mancha wetlands. *Cephalota circumdata imperialis* and *Myriochila melancholica melancholica*, present less dense populations, and also a more restricted local distribution, but they are nonetheless well distributed across the area. *Lophyra flexuosa flexuosa* and *Cicindela maroccana*, two widespread species in Central Spain (Ortuño and Toribio 1996), are scarce at La Mancha wetlands and their populations are represented by a low number of specimens. Two species, *Cephalota circumdata* and *Cephalota dulcinea*, are protected under Castilla – La Mancha legislation (620–CMA 20 Decreto 33/1998 Catálogo Regional de Especies Amenazadas de Castilla-La Mancha).

Most of the studied lakes and marshes are legally protected (http://ec.europa.eu/environment/nature/natura2000/db_gis/). Conservation efforts over the last decade have been directed at restoring the original ecological system, and also at eliminating the remains of human activity, particularly the removal and restoration of drainage channels, removal of urban waste, closure and restoration of village dumpsters, control of artificial flooding, and, more recently, restoration of native vegetation (the *albardinal* and the *Limonium* prairies), restoration of adjacent agricultural lands around the lakes, removal of salt- related semi-industrial activities, and road tracks removal and restoration (Fig. 13).

We have detected a series of threats that may affect the whole cicindelid (and other salt- associated animals) community. These threats are mostly derived from current agricultural practices around marshes, non-adequate use of trails and dirt roads, and insufficient regulation of water quality. Other threats might be derived from the restoration process itself, particularly the removal of abandoned semi-industrial infrastructures and trails.

Ploughing and cultivation of cereal, legumes, garlic or grapes, in fields adjacent to the lakes (Fig. 13f), and sheep grazing on the vegetated areas of lakes or around them (Fig. 13e) are still usual practices over most of the region. Consequences of these activities include the incorporation into the lake basin of large amounts of agricultural soil, drained from ploughed fields during the rainy periods, concentration of fertilizers or pesticides in the lake, washed from adjacent treated fields, direct changes in soil structure as sheep step on the salt shores (Fig. 13e), and nitrification and vegetation changes by sheep activity (Kiehl et al. 2001, Knisley 2011). Trails and dirt roads are used traditionally by agricultural vehicles (Fig. 13b). These slow moving vehicles pose little threat to tiger beetles, other than compacting a portion of the already compacted road. However, in recent years, some trails and roads have been heavily used by fast quads, and 4x4 vehicles, which no doubt have a direct impact on tiger beetle populations, either by direct killing (Fig. 12c) (Spomer and Higley 1993, Knisley 2011, Cornelisse 2013), or by habitat modification, including a clear increment in dust and erosion. Some lakes located near villages are receiving treated fresh water during most of winter and spring (García-Ferrer et al. 2003, Pérez and García 2004). This practice involves minor fluctuations in water levels, which seem to be favourable for species such as *Calomera littoralis littoralis* and *Myriochila melancholica melancholica*, which maintain healthy populations in these areas (Pedro Muñoz, pools at Camino de Villafranca southeast end) (Fig. 6d). On the other hand, the addition of freshwater reduces salinity, and might have a negative effect on other species, which in fact are not present in such systems.

Restoration practices might have a significant impact on the larger saline lakes that were subjected in the past to semi-industrial activities, predominantly salt extraction. Salt extraction industries modified the salt flats by creating evaporation pools, scattered deep pits, and some channels (Figs 6a, c and 13a, c, d). The removal of these man-made structures may represent a direct impact on some species that use the sides of the pools and the inclines of the pit sides and channels. These are places highly favoured by *Cephalota maura*, and sometimes used by *Cicindela campestris*, *Calomera littoralis* that *Myriochila melancholica*, and seem to add a component of structural diversity to the system. Mazzei et al. (2014) indicate that population densities of *Calomera littoralis* in disturbed habitats are lower than in non-disturbed ones, although for these authors disturbance is caused directly by human presence (beaches in summertime), rather than a consequence of ancient habitat modifications.

Closure and restoration of trails and roads around the lakes might pose a threat for species such as *Cephalota dulcinea*, that consistently use them for hunting and mating. Further studies are required to evaluate the role of these man-made structures for the species, and particularly whether they have any impact on larval development.

As general recommendations, we propose that the current practices of governmental buying of agricultural land around the lakes are absolutely necessary, both to avoid potentially dangerous agricultural practices and to create a buffer zone from other activities such as sheep grazing. Trails and minor roads must be closed for high risk sport practices (quads and 4x4 vehicles), while they can be used for other less aggressive activities. We recommend the maintenance of trails around lakes until their effect on populations of *Cephalota dulcinea* is properly evaluated. Old semi-industrial structures, evaporation pools, pits and channels, may be retained to increase structural diversity of the lakes. Finally, and especially considering the possible metapopulation system of *Cephalota dulcinea*, we consider necessary the implementation of legal protection for all salt marshes and saline lakes, of the occupation area of *Cephalota dulcinea*, including all minor entities that could act as migrational steps during recolonization events.

Our data support the status of protected species for *Cephalota circumdata*, a definite indicator of well-preserved salt lake flats, with relatively low population densities, and *Cephalota dulcinea*, an endemism to the salt marshes and lakes of La Mancha. A species appearing as a good indicator of protected halophytic prairies (*Limonium*, *Lygeum*) is *Cylindera paludosa*, but we do not consider necessary additional specific protection for this species.

## Supplementary materials

Supplementary material 1 Appendix Data type: Occurrence Explanation note: Field observations. This dataset is made available under the Open Database License (http://opendatacommons.org/licenses/odbl/1.0/). The Open Database License (ODbL) is a license agreement intended to allow users to freely share, modify, and use this Dataset while maintaining this same freedom for others, provided that the original source and author(s) are credited.Paula C. Rodríguez-Flores, Jorge Gutiérrez-Rodríguez, Ernesto F. Aguirre-Ruiz, Mario García-París
