# Supplementary material for: Salt lakes of La Mancha (Central Spain): A hot spot for tiger beetle (Carabidae, Cicindelinae) species diversity
Source: Zookeys. 2016 Feb 8;(561):63–103. doi: 10.3897/zookeys.561.6042 (PMC4768367; doi:10.3897/zookeys.561.6042)
Supplement: Supplementary material 1 — Appendix [file zookeys-561-063-s001.docx]

***Appendix***

Field observations. Detailed account of geo-referenced specimen data avalaible upon request from the senior author.

## Calomera littoralis littoralis

**Ciudad Real**

Alcázar de San Juan: Laguna de las Yeguas: 26-IV-2014 (n=19), 2-VIII-2014 (n=3)

Alcázar de San Juan: Laguna del Camino de Villafranca: 24-V-2014 (n=1), 11-VII-2014 (n=16), 27-VII-2014 (n=8), 2-VIII-2014 (n=7), 9-VIII-2014 (n=16)

Campo de Criptana: Laguna del Salicor: 28-VI-2014 (n=1), 8-VIII-2014 (n=10)

Pedro Muñoz: Laguna del Alcahozo: 6-VI-2014 (n=1), 8-VIII-2014 (n=3)

Pedro Muñoz: Laguna del Pueblo: 25-IV-2014 (n=2), 28-VI-2014 (n=32), 12-VII-2014 (n=17)

**Cuenca**

Carrascosa del Campo: Embalse: 15-VI-2014 (n=8)

Mota del Cuervo: Laguna de La Dehesilla: 16-V-2014 (n=21), 6-VI-2014 (n=16), 8-VIII-2014 (n=15)

Mota del Cuervo: Laguna de Manjavacas: 25-IV-2014 (n=3), 16-V-2014 (n=8), 28-VI-2014 (n=1), 8-VIII-2014 (n=13)

Mota del Cuervo: Laguna de Sánchez Gómez: 8-VIII-2014 (n=4)

Mota del Cuervo: Laguna del Alcahozo Chico: 16-V-2014 (n=24), 6-VI-2014 (n=15), 12-VII-2014 (n=3)

**Toledo**

Lillo: Altillo Chica: 2-VIII-2014 (n=2)

Lillo: Laguna del Longar: 11-VII-2014 (n=29), 2-VIII-2014 (n=53), 9-VIII-2014 (n=33), 23-VIII-2014 (n=18)

Miguel Esteban: La Laguna: 12-VII-2014 (n=9)

Quero: Laguna Grande: 26-IV-2014 (n=20), 24-V-2014 (n=2)

Villacañas: Laguna de Peña Hueca: 13-IV-2014 (n=5), 17-V-2014 (n=3), 11-VII-2014 (n=4), 2-VIII-2014 (n=2), 9-VIII-2014 (n=1)

Villacañas: Laguna de Tirez: 27-VI-2014 (n=2), 23-VIII-2014 (n=1), 24-X-2014 (n=1)

Villacañas: Laguna Larga: 27-VI-2014 (n=21), 9-VIII-2014 (n=3), 23-VIII-2014 (n=19)

Villafranca de Los Caballeros: Laguna de la Sal: 26-IV-2014 (n=2)

Villafranca de Los Caballeros: Laguna Grande: 20-VI-2014 (n=27), 27-VI-2014 (n=16), 11-VII-2014 (n=12), 2-VIII-2014 (n=8), 9-VIII-2014 (n=6)

***Cephalota (Cassolaia) maura***

**Ciudad Real**

Alcázar de San Juan: Laguna del Camino de Villafranca: 24-V-2014 (n=11)

Alcázar de San Juan: Laguna de las Yeguas: 20-VI-2014 (n=3)

Alcázar de San Juan: Camino de la Laguna de Pajares: 7-VI-2014 (n=9)

Alcázar de San Juan: Laguna de Pajares: 7-VI-2014 (n=1)

Campo de Criptana: Arroyo de San Marcos: 8-VI-2012 (n=21), 17-V-2014 (n=3)

# Cuenca

Belinchón: 15-VI-2014 (n=2)

Mota del Cuervo: Manjavacas: 21-VI-2014 (n=11), 28-VI-2014 (n=2), 12-VII-2014 (n=5)

# Toledo

Lillo: Laguna del Longar: 17-VI-2012 (n=10), 13-VI-2014 (n=4), 11-VII-2014 (n=3), 2-VIII-2014 (n=1)

Miguel Esteban: La Laguna: 12-VII-2014 (n=30)

Quero: Cerro San Cristóbal: 8-VI-2012 (n=18)

Villacañas: Laguna de Peña Hueca: 27-VI-2014 (n=1)

Villafranca de Los Caballeros: Laguna Grande: 20-VI-2014 (n=3), 27-VI-2014 (n=2), 11-VII-2014 (n=3)

***Cephalota (Taaenidia) circumdata imperialis***

**Ciudad Real**

Alcázar de San Juan: Laguna de las Yeguas: 20-VI-2014 (n=3), 2-VIII-2014 (n=3)

Campo de Criptana: Laguna de Salicor: 8-VIII-2014 (n=1)

Pedro Muñoz: Laguna del Alcahozo: 21-VI-2014 (n=8), 8-VIII-2014 (n=4)

# Toledo

Lillo: Laguna del Altillo Chica: 13-VI-2014 (n=33), 2-VIII-2014 (n=10)

Lillo: Laguna del Altillo Grande: 13-VI-2014 (n=11)

Lillo: Laguna del Longar: 2-VIII-2014 (n=2), 9-VIII-2014 (n=1)

Villacañas: Laguna de Peña Hueca: 13-VI-2014 (n=1), 11-VII-2014 (n=2), 2-VIII-2014 (n=1)

Villacañas: Laguna de Tirez: 20-VI-2014 (n=6), 27-VI-2014 (n=14), 23-VIII-2014 (n=1)

***Cephalota (Taaenidia) dulcinea***

**Ciudad Real**

Alcázar de San Juan: Camino de la Laguna de Pajares: 7-VI-2014 (n=37)

Alcázar de San Juan: Laguna de las Carros: 8-VI-2012 (n=19), 24-V-2014 (n=62), 21-VI-2014 (n=6)

Alcázar de San Juan: Laguna de las Yeguas: 17-V-2014 (n=44), 24-V-2014 (n=15), 20-VI-2014 (n=9)

Alcázar de San Juan: Laguna de Pajares: 7-VI-2014 (n=3)

Alcázar de San Juan: Laguna del Camino de Villafranca: 24-V-2014 (n=2)

Campo de Criptana: Laguna de Salicor: 8-VI-2012 (n=1)

Pedro Muñoz: Laguna del Alcahozo: 16-V-2014 (n=1), 6-VI-2014 (n=7), 21-VI-2014 (n=2)

**Cuenca**

El Hito: Laguna del Hito: 15-VI-2014 (n=29)

Mota del Cuervo: Laguna de La Dehesilla: 6-VI-2014 (n=25), 21-VI-2014 (n=5)

Mota del Cuervo: Laguna de Sánchez Gómez: 16-V-2014 (n=27), 6-VI-2014 (n=64), 28-VI-2014 (n=4)

**Toledo**

Lillo: Laguna del Altillo Chica: 7-VI-2014 (n=3), 13-VI-2014 (n=9)

Lillo: Laguna del Altillo Grande: 7-VI-2014 (n=24), 13-VI-2014 (n=3)

Lillo: Laguna del Longar: 15-VI-2012 (n=6), 17-VI-2012 (n=14), 23-V-2014 (n=13), 13-VI-2014 (n=26)

Quero: Cerro San Cristóbal: 24-V-2014 (n=23)

Quero: Laguna del Taray: 7-VI-2014 (n=11)

Villacañas: Laguna de Peña Hueca: 17-V-2014 (n=9), 23-V-2014 (n=8), 13-VI-14 (n=7), 27-VI-2014 (n=5)

Villacañas: Laguna de Tirez: 17-VI-2012 (n=3), 24-V-2014 (n=12), 20-VI-2014 (n=13), 27-VI-2014 (n=9)

Villafranca de Los Caballeros: Laguna de la Sal: 24-V-2014 (n=6)

Villafranca de Los Caballeros: Laguna Grande: 27-V-2014 (n=1)

***Cicindela (Cicindela) campestris campestris***

**Ciudad Real**

Alcázar de San Juan: Laguna de las Yeguas: 26-IV-2014 (n=1)

Campo de Criptana: Arroyo de San Marcos: 8-VI-2012 (n=10), 17-V-2014 (n=4)

Campo de Criptana: Laguna del Salicor: 13-IV-2014 (n=10)

Pedro Muñoz: Laguna de Navalafuente: 25-IV-2014 (n=1)

**Cuenca**

Las Mesas: Laguna del Taray Chico: 25-IV-2014 (n=2), 16-V-2014 (n=1)

Mota del Cuervo: Laguna de La Dehesilla: 16-V-2014 (n=12)

Mota del Cuervo: Laguna de Sánchez Gómez: 16-V-2014 (n=1)

Mota del Cuervo: Laguna del Alcahozo Chico: 25-IV-2014 (n=10), 16-V-2014 (n=18)

**Toledo**

Lillo: Laguna del Longar: 13-IV-2014 (n=8), 23-V-2014 (n=1)

Quero: Cerro San Cristóbal: 8-VI-2012 (n=1),

Villacañas: Laguna de Tirez: 13-IV-2014 (n=6), 24-X-2014 (n=3)

***Cicindela (Cicindela) maroccana***

**Toledo**

Villacañas: Laguna de Peña Hueca: 13-IV-2014 (n=2)

Villacañas: Laguna de Tirez: 13-IV-2014 (n=4)

Villafranca de Los Caballeros: Laguna de la Sal: 26-IV-2014 (n=2)

***Cylindera (Cylindera) paludosa***

**Ciudad Real**

Alcázar de San Juan: Camino de la Laguna de Pajares: 7-VI-2014 (n=3)

Alcázar de San Juan: Laguna de las Yeguas: 17-V-2014 (n=4), 20-V-2014 (n=4), 24-V-2014 (n=1)

Alcázar de San Juan: Lagunas del Gigüela: 24-V-2014 (n=7)

Campo de Criptana: Arroyo de San Marcos: 17-V-2014 (n=1)

**Cuenca**

Mota del Cuervo: Laguna de La Dehesilla: 6-VI-2014 (n=2)

Mota del Cuervo: Laguna del Alcahozo Chico: 16-V-2014 (n=1), 6-VI-2014 (n=3)

Lillo: Laguna del Altillo Chica: 7-VI-2014 (n=10), 13-VI-2014 (n=6)

Lillo: Laguna del Altillo Grande: 23-V-2014 (n=6), 7-VI-2014 (n=10)

Lillo: Laguna del Longar: 15-VI-2012 (n=16), 17-VI-2012 (n=21), 23-V-2014 (n=7), 9-VIII-2014 (n=1)

Quero: Laguna del Taray: 21-VI-2014 (n=1)

Villacañas: Laguna de Tirez: 17-VI-2012 (n=4)

Villacañas: Laguna Larga: 17-VI-2012 (n=1)

***Lophyra (Lophyra) flexuosa flexuosa***

**Ciudad Real**

Campo de Criptana: Laguna del Salicor: 8-VI-2012 (n=5)

**Cuenca**

Mota del Cuervo: Laguna del Alcahozo Chico: 16-V-2014 (n=3)

## Myriochila (Myriochila) melancholica melancholica

**Ciudad Real**

Alcázar de San Juan: Laguna del Camino de Villafranca: 11-VII-2014 (n=1), 27-VII-2014 (n=2), 9-VIII-2014 (n=8)

Pedro Muñoz: Laguna del Pueblo: 28-VI-2014 (n=8), 12-VII-2014 (n=6)

**Toledo**

Lillo: Laguna del Longar: 11-VII-2014 (n=6)

Villacañas: Laguna de Tirez: 27-VI-2014 (n=4)
